# Quantitative proteomic analysis of Parkin substrates in *Drosophila* neurons

**DOI:** 10.1186/s13024-017-0170-3

**Published:** 2017-04-11

**Authors:** Aitor Martinez, Benoit Lectez, Juanma Ramirez, Oliver Popp, James D. Sutherland, Sylvie Urbé, Gunnar Dittmar, Michael J. Clague, Ugo Mayor

**Affiliations:** 1 Department of Biochemistry and Molecular Biology, University of the Basque Country (UPV/EHU), Leioa, Bizkaia Spain; 2 Functional Genomics Unit, CIC bioGUNE, Derio, Spain; 3 Department of Cellular and Molecular Physiology, Institute of Translational Medicine, University of Liverpool, Liverpool, UK; 4 Max Delbrück Center for Molecular Medicine, Berlin, Germany; 5 Ikerbasque, Basque Foundation for Science, Bilbao, Bizkaia Spain; 6 Department of Oncology, Luxembourg Institute of Health, Luxembourg City, Luxembourg

**Keywords:** Parkin (PARK2), Parkin substrates, Ubiquitination, VPS35, Neurodegeneration, In vivo, *Drosophila melanogaster*, Parkinson’s Disease (PD), Alzheimer’s Disease (AD), Label Free Quantification (LFQ)

## Abstract

**Background:**

Parkin (PARK2) is an E3 ubiquitin ligase that is commonly mutated in Familial Parkinson’s Disease (PD). In cell culture models, Parkin is recruited to acutely depolarised mitochondria by PINK1. PINK1 activates Parkin activity leading to ubiquitination of multiple proteins, which in turn promotes clearance of mitochondria by mitophagy. Many substrates have been identified using cell culture models in combination with depolarising drugs or proteasome inhibitors, but not in more physiological settings.

**Methods:**

Here we utilized the recently introduced BioUb strategy to isolate ubiquitinated proteins in flies. Following Parkin Wild-Type (WT) and Parkin Ligase dead (LD) expression we analysed by mass spectrometry and stringent bioinformatics analysis those proteins differentially ubiquitinated to provide the first survey of steady state Parkin substrates using an in vivo model. We further used an in vivo ubiquitination assay to validate one of those substrates in SH-SY5Y cells.

**Results:**

We identified 35 proteins that are more prominently ubiquitinated following Parkin over-expression. These include several mitochondrial proteins and a number of endosomal trafficking regulators such as v-ATPase sub-units, Syx5/STX5, ALiX/PDCD6IP and Vps4. We also identified the retromer component, Vps35, another PD-associated gene that has recently been shown to interact genetically with *parkin*. Importantly, we validated Parkin-dependent ubiquitination of VPS35 in human neuroblastoma cells.

**Conclusions:**

Collectively our results provide new leads to the possible physiological functions of Parkin activity that are not overtly biased by acute mitochondrial depolarisation.

**Electronic supplementary material:**

The online version of this article (doi:10.1186/s13024-017-0170-3) contains supplementary material, which is available to authorized users.

## Background

Parkinson’s Disease (PD) is the most common form of Parkinsonism and the second most common neurodegenerative disease after Alzheimer’s. PD patients display resting tremor, rigidity and postural disability, usually accompanied by other non-motor symptoms. Phenotypically, PD is mainly characterized by loss of dopaminergic neurons in the substantia nigra and the presence of Lewy bodies [1, 2]. PD has been classically considered a sporadic disease linked to aging with an unknown aetiology. However, in about 10% of the cases, mutations in specific genes cause Familial forms of PD. These genes show Mendelian inheritance and can be classified as either autosomal dominant (SNCA, LRRK2, VPS35) or autosomal recessive (PARK2, PARK7, PINK1, ATP13A2, FBXO7, PLA2G6, DNAJC6, SYNJ1). Moreover, recent exome sequencing and cohort genome-wide association studies (GWAS) have identified several other risk factor genes associated with sporadic PD and other Parkinsonian syndromes [3–6]. The set of genes implicated in PD encode for proteins involved in mitochondrial homeostasis, autophagy, endo-lysosomal trafficking, Ca^+2^ homeostasis and dopamine homeostasis [7–10]. However, the exact pathophysiological mechanisms leading to the disease are not yet clear. At the moment there is no effective biomarker for the diagnosis of PD, which can only be determined by postmortem brain analysis [11].

Amongst PD related genes, mutations in Parkin (PARK2) and PTEN-induced kinase 1 (PINK1) cause early-onset Familial PD [12, 13]. Parkin is a RING between RING (RBR) E3 ubiquitin ligase, which conveys the transfer of ubiquitin onto selected substrate proteins [14, 15]. Parkin null *Drosophila melanogaster* display Parkinsonian-like phenotypes including reduced life span, climbing and flying disability, sterility, mitochondrial defects and dopaminergic neurodegeneration [16]. Genetic studies in *Drosophila* established that *pink1* acts upstream of *parkin* to maintain mitochondrial integrity [17, 18]. Upon mitochondrial depolarization PINK1 accumulates at the Outer Mitochondrial Membrane (OMM), where it phosphorylates both ubiquitin and the Ubiquitin-like (UBL) domain of Parkin to recruit and activate latent Parkin ubiquitin ligase activity [19–25]. Activated Parkin ubiquitinates several OMM proteins and promotes both proteasome-dependent degradation of specific proteins and mitophagy, a specialised type of autophagy where the whole mitochondrion is engulfed into autophagosomes [26–28].

PINK1 and Parkin are widely considered neuroprotective and different studies have shown that PINK1/Parkin over-expression can protect against cell death in a number of contexts in vitro and in vivo [29]. Therefore it has been proposed that drugs promoting PINK1/Parkin - dependent mitophagy could serve as effective treatments for PD. However, recent evidence demonstrates that excessive Parkin over-expression results in sensitization to cell death using in vitro [30–32] and in vivo models [33].

It is essential to identify physiologically relevant Parkin substrates to understand the pathways leading to PD in order to develop a treatment. A considerable number of proteins have been reported to be Parkin substrates but most of the work has relied on cultured cells, mainly of epithelial origin, usually upon treatment with mitochondrial depolarising agents [27, 34–38]. Here we extend this approach by performing a high throughput mass spectrometry proteomic study of Parkin substrates in vivo. We have utilised a fly model expressing constitutively biotinylated ubiquitin [39–43] to purify proteins ubiquitinated by Parkin in *Drosophila* neurons. Our study identifies both established and novel Parkin substrates.

## Methods

### DNA construction

*Drosophila park* gene was amplified from a *Drosophila* cDNA library (DGC realease 1.0, Berkeley Drosophila Genome Project) and FLAG-tag cloned at its 5’-end using the FLAG-*parkin-Fw* (GCCCTCGAGATGGATTACAAGGATGATGACGATAAGATGAGTTTTATTTTTAAATTTATTGCCAC) and *parkin*-*Rv* (GCCTCTAGATTAGCCGAACCAGTGGGCTCC) primers. This construct was then inserted into a pUASattb vector between the *XhoI* and *XbaI* sites. Ligase-dead FLAG-Parkin (Parkin^LD^) was generated by mutating the C449 to S using the QuikChange Site-Directed Mutagenesis Kit (Stratagene) according to manufacturer’s instructions. The primers used for mutagenesis were *C449S-Fw* (GGAGCGAGATGGCGGT***A***GCATGCACATGGTCTGCACACG) and *C449S-Rv* (CGTGTGCAGACCATGTGCATGC***T***ACCGCCATCTCGCTCC). Untagged human Parkin and Parkin (C431S) were amplified from pcDNA3.1(+)-HA-Parkin and pcDNA3.1(+)-HA-Parkin(C431S) respectively with primers forward (GCCGAAGCTTAACCATGATAGTGTTTGTCAGG) and reverse (AGTCTAGACTACACGTCGAACCAGTGGTCCTGGG). PCR products were inserted into pcDNA3.1(+) between HindIII and XbaI.

### Antibodies

The following antibodies were used against *Drosophila* proteins*:* goat anti-biotin-horseradish peroxidase (HRP) conjugated antibody (Cell Signalling); chicken polyclonal anti-BirA antibody (Sigma); rabbit polyclonal anti-Parkin antibody [44]; mouse monoclonal anti-Syx1A antibody (DSHB); rabbit polyclonal anti-RdhB [45]; rabbit polyclonal anti-ArgK [46]; rabbit polyclonal anti-Vps4 [47]; rabbit polyclonal anti-Fax antibody (a gift from Eric Liebl); rabbit polyclonal anti-Ubiquitin antibody (Sigma). The following antibodies were used against Human proteins: goat polyclonal anti-VPS35 antibody (Abcam); mouse monoclonal anti-Cleaved Parp-85 fragment (Cell Signaling); mouse monoclonal anti-Parkin (Santa Cruz); rabbit polyclonal anti-PINK1 (Novus Biologicals); rabbit polyclonal anti-Miro1 (Sigma); rabbit polyclonal anti-Tim44 (Sigma); rabbit polyclonal anti-Tom20 (Sigma); mouse monoclonal (Abcam) and rabbit polyclonal (Sigma) anti-Actin. For monitoring the GFP pull-downs the following antibodies were used: monoclonal mouse anti-GFP antibody (Roche) and monoclonal mouse anti-Flag M2-HRP conjugated antibody (Sigma). Anti-mouse, rabbit and chicken HRP labelled secondary antibodies (Jackson ImmunoResearch Laboratories) and anti-guinea pig (Invitrogen) were used; and anti-mouse, rabbit and sheep IR 680 and IR800-coupled antibodies (LI-COR Biosciences).

### Drosophila stocks

*UAS-BirA* and *UAS-(* ^*Bio*^ *Ub)*_*6*_ *-BirA* [39] and their recombination with *GMR-GAL4* flies for the study of ubiquitin proteomics has been previously described [43]. FLAG-tagged Parkin wild-type (Parkin^WT^) and Parkin^LD^ flies were generated at Bestgene using the pUASattb constructs described above. Both *UAS-Parkin* ^*WT*^ *and UAS-Parkin* ^*LD*^ lines were independently crossed with *GMR-GAL4,UAS-(* ^*Bio*^ *Ub)*_*6*_ *-BirA* to finally generate: *GMR-GAL4,UAS-(* ^*Bio*^ *Ub)*_*6*_ *-BirA/CyO;UAS-Parkin* ^*WT*^ and *GMR-GAL4,UAS-(* ^*Bio*^ *Ub)*_*6*_ *-BirA/CyO;UAS-Parkin* ^*LD*^. *GMR-GAL4/CyO;UAS-BirA/TM6* and *GMR-GAL4,UAS-(* ^*Bio*^ *Ub)*_*6*_ *-BirA/CyO* flies were additionally used as controls. *UAS-GFP, elav-GAL4, GMR-GAL4, Tub-GAL4, Da-GAL4, Ple-GAL4* flies were obtained from Bloomington *Drosophila* Stock Center. *UAS-GFP* ^*CL1*^ flies were obtained from [48] and *park* ^*25*^ */TM6b GFP-w* ^*+*^ and *UAS-park* were obtained from [16]. Flies were grown in 12 h light-dark cycles at 25 °C and were fed with wheat flour and yeast food (1% agar, 5.5% dextrose, 3.5% wheat flour, 5% yeast, 0.25% Nipagen, 0.4% Propionic acid and 0.02% Benzalkonium Chloride in distilled H_2_O).

### Climbing assay

Flies of indicated ages and genotypes were anesthetised with CO_2_ on a pad, and 20 flies (10 male and 10 female) were randomly selected. After an hour of recovery, flies were transferred to a climbing vial and ability to climb was scored as followed. Flies were gently tapped to the bottom and the number of flies that reached the 10 cm mark at 30 s was counted three times, with 30 s interval.

### Survival assay

One hundred newborn flies of the indicated genotypes were maintained in wheat flour and yeast food in 12 h light-dark cycles at 25 °C. The vials were changed every 2–3 days and the number of flies alive was counted.

### Fly head extract preparation and cell lysis

Six heads (three males and three females) of adult flies were cut and homogenised in 60 μL of 4x Laemmli buffer with DTT. Samples were centrifuged 1 min at maximum spin and the supernatant was recovered. Cells were harvested using “Hot Lysis buffer” (2% SDS, 50 mM NaF and 1 mM EDTA at 110 °C; Fig. 8). Protein concentrations were determined by BCA protein assay (Pierce).

### Cell culture, siRNA knockdown and transfection

hTERT-RPE1-Parkin cells [32, 49] were cultured in Dulbecco’s modified Eagle’s medium (DMEM)/F12 with 10% FBS, 1% non-essential amino acids and 1% penicillin/streptomycin. Reverse transfection was performed using Lipofectamine RNAi-MAX (Invitrogen) and carried out for 72 h according to manufacturer’s instruction. Further 165 h double-hit knockdown was executed by transfecting again previously siRNA treated cells at 72 h. Cells were transfected with the following siRNA at a final concentration of 20nM: Non-Targeting siRNA oligo #1 (NT1) (ONTARGETplus: NT1; 5’-UGGUUUACAUGTCGACUAA-3’) and VPS35 (SMARTpool ONTARGETplus VPS35 siRNA oligo#5: 5’-GAACAUAUUGCUACCAGUA-3’; oligo#6: 5’-GAAAGAGCAUGAGUUGUUA-3’; oligo#7: 5’-GUUGUAAACUGUAGGGAUG-3’; oligo#8: 5’-GAACAAAUUUGGUGCGCCU-3’) from Dharmacon.

Human neuroblastoma SH-SY5Y cells were cultured in DMEM supplemented with 10% FBS (Thermo Scientific) and 1:100 penicillin/streptomycin at 37 °C. 300,000 cells were seeded in a 6 well-plate and incubated overnight under serum starvation. The following day, medium was removed, replaced by fresh DMEM and cells were transfected with 1 μg of FLAG-Ub, 1 μg of YFP-VPS35 together with 1 μg of pcDNA3.1 (control) or 1 μg of hParkin or 1 μg of hParkin (C431S) using Lipofectamine 3000 (Invitrogen) for 72 h according to manufacturer’s instruction.

### GFP beads pull-down assay

Transfected cells were lysed in 500 μl of lysis buffer [50 mM Tris–HCl pH 7.5, 150 mM NaCl, 1 mM EDTA, 0.5% Triton, 1 × Protease Inhibitor cocktail (Roche Applied Science), 50 mM *N*-ethylmaleimide (NEM, from Sigma)] and collected for centrifugation at 14,000 × *g* for 10 min. The supernatant was mixed with 25 μL of GFP-Nanotrap beads (Chromotek GmbH) that had been pre-washed with dilution buffer (10 mM Tris–HCl pH 7.5, 150 mM NaCl, 0.5 mM EDTA, 1 × Protease Inhibitor cocktail, 50 mM NEM). The mixture was incubated at RT for 150 min with gentle rolling and centrifuged for 2,700 × *g* for 2 min. The supernatant was removed and the beads washed once with dilution buffer, three times with washing buffer (8 M Urea, 1% SDS in PBS) and once with 1% SDS in 1× PBS. The bound proteins were eluted with sample loading buffer (250 mM Tris–HCl pH 7.5, 40% glycerol, 4% SDS, 0.2% BPB) by heating at 95 °C for 10 min. Eluted samples were run into 4–15% Tris–Glycine gels.

### Western blotting and silver staining

For fly samples the amount of sample loaded largely was equivalent to one head per lane but was optimized for each antibody. Generally, 4–15% gradient TGX gels (Bio-Rad) were used and proteins were transferred to PVDF membranes using the iBlot system (Invitrogen). Following primary and secondary antibody incubation, membranes were developed using ECL kit (Biorad Clarity). Alternatively, western blots were scanned using the LI-COR Odyssey system (LI-COR Biosciences). Silver staining was performed with a SilverQuest kit (Invitrogen) following the manufacturer’s instructions. Dual-colour westerns were prepared by assigning independent colour channels to two independent westerns developed in the same membrane.

### ^Bio^Ub pulldown

The ^Bio^Ub pulldown was performed as previously described [39, 40, 43] using *GMR-GAL4,UAS-(* ^*Bio*^ *Ub)*_*6*_ *-BirA/CyO; UAS-Parkin* ^*WT*^ *, GMR-GAL4,UAS-(* ^*Bio*^ *Ub)*_*6*_ *-BirA/CyO;UAS-Parkin* ^*LD*^ *, GMR-GAL4/CyO;UAS-BirA/TM6 and GMR-GAL4, UAS-(* ^*Bio*^ *Ub)*_*6*_ *-BirA/CyO* flies. Briefly, 2–5 day old adult flies were fragmented by flash freezing in liquid nitrogen and shaking. A pair of sieves with a nominal cut-off of 710 and 425 μm was used to separate the heads from the rest of the fragments. About 0.5 g of heads were homogenized in 2.9 mL Lysis buffer (8 M Urea, 1% SDS and 50 mM N-ethylmaleimide in PBS, including a protease inhibitor mixture from Roche Applied Science). 250 μL of NeutrAvidin-agarose beads (Thermo Scientific) were used to purify ^Bio^Ubiquitinated material and after stringent washes with a succession of buffers containing 8.4 M urea, 6 M Gdn-HCl, 1 M NaCl and 2%SDS, beads were finally eluted with 125 μL Elution Buffer (4X Laemmli Buffer and 100 mM of DTT). Recovered volume was ~150 μL with an average yield of 20–40%.

### Mass spectrometry

Eluted samples from three different experiments were briefly run on a SDS-PAGE, which was then cut in four slices, to separate the avidin band from the other proteins in the gel. Proteins were digested using automated in-gel digestion protocol [50]. The digested peptides were cleaned-up on Stage-tips [51]. The eluted peptides were resuspended in 0.1% formic acid and separated on a 15 cm reverse phase column (75 μm inner diameter, 3 μm reprosil beads, Dr. Maisch, GmbH, in house packed) using 5 to 50% acetonitrile gradient (VWR). Peptide ionization was performed on a Proxeon ionsource and sprayed directly into the mass spectrometer (Q-Exactive, Thermo Scientific). The MS was recorded with mass resolution of 60000, while the MS2 spectra were collected with a resolution of 35000 using a fixed fill time of 120 ms. For the analysis of the recorded mass spectra the MaxQuant software package (version 1.2.2.5) was used with 1% FDR for both peptides and proteins [52]. Searches were performed using the Andromeda search engine against the Uniprot *Drosophila melanogaster* database (downloaded 15.08.2015). As a fixed modification, cysteine carbamidomethylation was selected and as variable modifications, methionine oxidation, protein N-terminal acetylation and di-glycine addition on the ε-aminogroup of lysines. Two missed trypsin (full specificity) cleavages were allowed and the MaxQuant label free quantification for proteins was activated. The identified ubiquitination sites were checked visually using the Max-Quant View program and identification artefacts were removed as previously described [53].

### Ubiquitin chain linkage quantification

Samples from three technical replicates were loaded on an 8% stacking gel and proteins compressed in one band. Proteins were digested in-gel using trypsin, and purified as described above. The peptides were separated on a 25 cm reverse phase column (75 μm inner diameter, 3 μm reprosil beads, Dr. Maisch, GmbH, in house packed) using 5 to 50% acetonitrile gradient (VWR). The peptides were ionized on a Nano3 ion source (ABSciex) and directly sprayed into a QTRAP 5500 triple-quadrupole mass spectrometer, run in MRM mode to detect branched ubiquitin peptides and their synthetic isotopically labelled counterparts [54, 55]. Each sample was injected as technical triplicates. Two transitions for each peptide, i.e., K6, K11, K48, K63, were acquired. After the measurement peaks were integrated and light/heavy ratios calculated using the Skyline software package [56] reflecting the abundance of the linkage type compared to the internal heavy standard.

### Bioinformatic and statistical analysis

Non-ubiquitinated/background proteins were excluded by a 4-fold change threshold of the average LFQ intensity between ^Bio^Ub, ^Bio^Ub + Parkin^WT^ or ^Bio^Ub + Parkin^LD^ against the BirA sample. LFQ intensity values of ubiquitinated proteins were imported to Perseus software (http://www.perseus-framework.org/) and the imputation tool was used to replace missing values. Subsequently, values were grouped in categories and two T-tests were performed: ^Bio^Ub + Parkin^WT^ *vs* ^Bio^Ub and ^Bio^Ub + Parkin^WT^ *vs* ^Bio^Ub + Parkin^LD^. Proteins with a *p*-value smaller than 0.05 and a fold change bigger or smaller than 2 in any of these two tests were selected for further filtering. Proteins with less than two unique peptides in all the three ^Bio^Ub + Parkin^WT^ (enriched) and ^Bio^Ub + Parkin^LD^ (reduced) were excluded. To further exclude false positives, all unique peptide profiles of these proteins were analysed. Peptides that appeared only in one condition were excluded and the average peptide intensity was calculated for each peptide in each condition. On the one hand, average of all peptide intensities between ^Bio^Ub + Parkin^WT^ *vs* ^Bio^Ub or ^Bio^Ub + Parkin^LD^ were calculated and only the proteins whose total fold change was bigger (enriched) or smaller (reduced) than two were further selected. On the other hand, the average of all fold changes of peptide intensities between ^Bio^Ub + Parkin^WT^ *vs* ^Bio^Ub or ^Bio^Ub + Parkin^LD^ minus the SEM (Standard Error of the Mean) was required to be bigger or smaller than two. Only the proteins that successfully passed all the tests were selected as: Parkin substrates (35) and less ubiquitinated in a Parkin - dependent manner (2).

A two sample T-Test for Parkin proteomics was performed using Perseus software applying default parameters: t-test; S0; Side = both; threshold *p*-value = 0,05. Imputation tool was used as default: width = 0,3; down shift = 1,8; mode = separately for each column. Other statistical analysis was performed with Graphpad Prism6. For the GO Term identification g:Cocoa tool of g:Profiler web server was used using archive “Ensembl 79, Ensembl Genomes 26 (r1395)”. *p*-values are indicated as *p ≤ 0.05, **p ≤ 0.01 and ***p ≤ 0.001. Functional connectivity analysis was performed using STRING (http://string-db.org/) web tool.

For GFP pull-downs protein bands were analysed and quantified by densitometry using ImageJ software. Statistical significance in Western blotting quantification was evaluated using an analysis of variance (ANOVA) complemented by Tukey’s honest significance difference test (Tukey’s HSD) performed in GraphPad PRISM software. Statistical significance levels are annotated as ns, not significant; **, *p* < 0.01 (mean ± SEM, *n* = 4).

## Results

### Excessive Parkin activity in developing neurons causes climbing defects and reduced life span

**Fig. 1:**
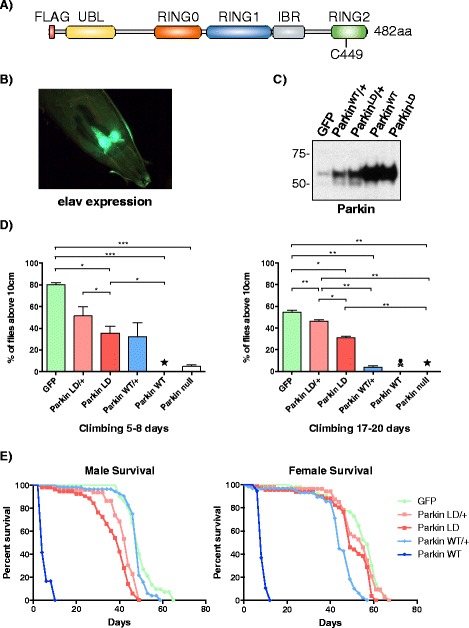
Parkin over-expression in developing neurons causes severe climbing defects and premature death in *Drosophila melanogaster*. **a** Domain structure of *Drosophila melanogaster* Parkin, cloned with a N-terminal FLAG tag. The catalytic cysteine (C449), marked with an *asterisk*, was mutated to serine to generate a Ligase-Dead (LD) Parkin mutant. **b** L3 larva showing GFP over-expression pattern in developing neurons using the *elav-GAL4* driver. **c** Anti-Parkin western blot showing Parkin levels in flies over-expressing one copy (Parkin^WT^/+ and Parkin^LD^/+) or two copies of FLAG-tagged WT (Parkin^WT^) and LD Parkin (Parkin^LD^), using *elav-GAL4*. GFP over-expression was used as a control. Four-fold less sample was loaded for Parkin^WT^ and Parkin^LD^. **d** Climbing assay of stated genotypes at 5–8 and 17–20 days after eclosion. Parkin null (*park* ^*25*^) flies were used as a negative control. Stars indicate 0% climbing ability and a skull represents complete death at the particular time point. Three technical replicates were performed with three biological replicates of each genotype and age. Columns represent average values and error bars show standard error of the mean (SEM). **e** Survival assay of stated genotypes. Survival graphs represent % of alive flies at each time point. Statistical significance was assessed by two-tailed paired Student’s-t test. *Asterisk*(s) indicate significance, *p ≤ 0.05, **p ≤ 0.01 and ***p ≤ 0.001. Complete genotypes of flies used: *elav-GAL4,UAS-GFP/CyO* (GFP), *elav-GAL4/CyO;UAS-Parkin* ^*WT*^ */TM6* (Parkin^WT^/+), *elav-GAL4/CyO;UAS-Parkin* ^*LD*^ */TM6* (Parkin^LD^/+), *elav-GAL4/CyO;UAS-Parkin* ^*WT*^ (Parkin^WT^), *elav-GAL4/CyO;UAS-Parkin* ^*LD*^ (Parkin^LD^) *and park* ^*25*^ (Parkin null)

In order to study Parkin E3 ubiquitin ligase activity in *Drosophila* neurons, we over-expressed FLAG-Parkin^WT^ (Fig. 1a) and a catalytically inactive or ligase-dead mutant, FLAG-Parkin^LD^, using the neuronal specific *elav-GAL4* driver (Fig. 1b). As expected, heterozygous FLAG-Parkin flies expressed lower Parkin levels compared to homozygous Parkin flies (Fig. 1c, Additional file 1: Figure S1A). Neuronal over-expression of Parkin^WT^ during *Drosophila* development compromises climbing ability of the fly in an age and dose-dependent manner, mirroring the phenotype of Parkin null flies (Fig. 1d, Additional file 1: Figure S1B). Expression of a ligase-dead form of Parkin generates a much milder climbing impairment. The N-terminal FLAG tag did not contribute to this behaviour as both untagged Parkin^WT^ and 8HA-Parkin^WT^ flies display the same phenotype (Additional file 1: Figure S1C). High-level expression of Parkin^WT^ also led to a decreased life span (Fig. 1e) and, in distinction to Parkin^LD^ flies, Parkin^WT^ flies displayed a Parkinsonian phenotype, suffering from tremors and spontaneous spasms (Additional file 12: Video S1).

### Parkin substrate identification in adult Drosophila neurons

**Fig. 2:**
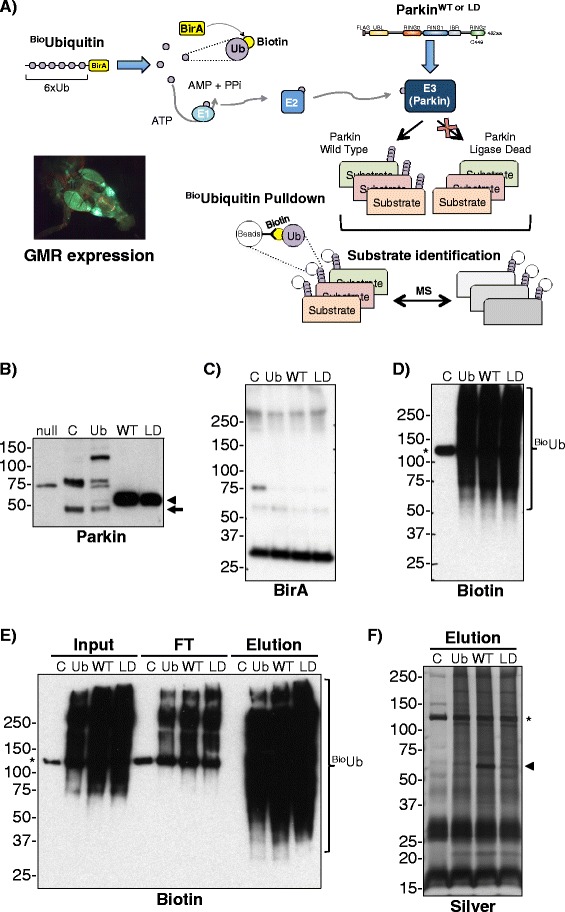
Proteomic analysis of Parkin substrates in *Drosophila melanogaster* neurons. **a** Scheme of the strategy used to identify proteins ubiquitinated by Parkin in *Drosophila* neurons. FLAG-tagged Parkin WT or LD flies also expressed biotin modified ubiquitin (^Bio^Ub) in *Drosophila* photoreceptors using *GMR-GAL4* driver (image shows GFP expression with *GMR-GAL4* in an adult fly head). ^Bio^Ubiquitinated material was purified using Neutravidin beads and isolated material was analysed by Mass Spectrometry (MS). Ubiquitinated proteins enriched in Parkin WT over-expressing neurons were then identified based both on protein LFQ levels and peptide intensities. **b** Anti-Parkin immunoblotting shows Parkin levels in head extracts of *GMR-GAL4/CyO;UAS-BirA/TM6* (C), *GMR-GAL4,UAS-(* ^*Bio*^ *Ub)*_*6*_ *-BirA/CyO* (Ub), *GMR-GAL4,UAS-(* ^*Bio*^ *Ub)*_*6*_ *-BirA/CyO;UAS-Parkin* ^*WT*^ (WT) and *GMR-GAL4,UAS-(* ^*Bio*^ *Ub)*_*6*_ *-BirA/CyO;UAS-Parkin* ^*LD*^ (LD) flies. Parkin null (*park* ^*25*^) flies were used as a negative control. WT and LD samples were diluted 100 times. **c** Anti-BirA western blot of C, Ub, WT and LD heads extracts. **d** Anti-Biotin western blot of C, Ub, WT and LD heads extracts. **e** C, Ub, WT, LD fly heads were subjected to ^Bio^Ub pulldown. Input (1/100), Flowthrough (FT, 1/100) and Elution (1/400) were immunoblotted for biotin. **f** Silver staining of C, Ub, WT, LD ^Bio^Ub pulldown elutions (undiluted). In panels B through F, arrowheads indicate ectopic Parkin; arrow indicates endogenous Parkin; smear highlighted by the brace represents ^Bio^Ubiquitinated material; and the *asterisk* shows an endogenously biotinylated protein

We recently introduced a pipeline for the identification of ubiquitinated proteins in a fly model that constitutively expresses biotinylated ubiquitin (^Bio^Ub). Here, we have applied this approach to identify physiologically relevant Parkin substrates in adult *Drosophila* neurons (Fig. 2a). ^Bio^Ubiquitin together with either Parkin^WT^ (WT) or Parkin^LD^ (LD) was over-expressed in *Drosophila* photoreceptors, a specialised type of neuron, using *GMR-GAL4* driver (Fig. 2b). Flies over-expressing just BirA (C) and ^Bio^Ubiquitin (Ub) were used as background control and steady state ubiquitination control respectively. Endogenous deubiquitinating enzymes (DUBs) digest the ^Bio^Ubiquitin construct releasing BirA (Fig. 2c) and ubiquitin. Each ubiquitin contains a short N-terminal sequence, which is then recognised and biotinylated by BirA, resulting in a biotin-tagged ubiquitin (^Bio^Ub) that is efficiently handled by the cascade of ubiquitin conjugating enzymes (Fig. 2d) [39–43]. ^Bio^Ub conjugates were isolated on Neutravidin beads (Fig. 2e, f) and subsequently identified by Mass Spectrometry (MS).

### Mass Spectrometry analysis of Parkin - dependent ubiquitination identified novel putative Parkin substrates in Drosophila neurons

**Fig. 3:**
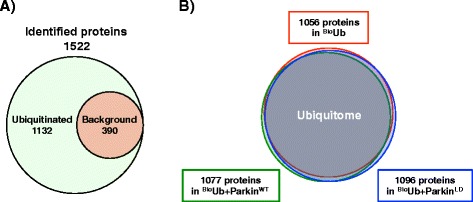
Characteristics of the data set. **a** Three independent ^Bio^Ub pulldowns were performed for each condition generating 1522 protein identifications by mass spectrometry of which 1132 were considered to be ubiquitinated by imposing a threshold of four-fold change in LFQ intensity relative to BirA control. **b** Venn diagram shows the overlap between proteins identified as ubiquitinated under different conditions, indicating that Parkin expression does not dramatically alter the global ubiquitome

Three independent experiments were performed for each condition (Additional file 2: Figure S2A), generating 1522 protein identifications by mass spectrometry (Additional file 9: Table S1). After background subtraction of the BirA control, 1132 proteins were classed as ubiquitinated by virtue of their enrichment (Fig. 3a). Most of the ubiquitinated proteins were identified in each of the three different conditions (Fig. 3b), suggesting that the ectopic expression of Parkin does not perturb the overall global ubiquitin economy. In fact, the number of proteins detected to be ubiquitinated in each pulldown was very similar across all samples (Additional file 3: Figure S3A) and Label Free Quantification (LFQ) values of independent pulldowns were highly correlated (Additional file 4: Figure S4). For proteins not directly quantitated in one experimental condition (LFQ = 0) we used the Perseus imputation tool to assign an arbitrary value within the lower detection range (Additional file 3: Figure S3B).

**Fig. 4:**
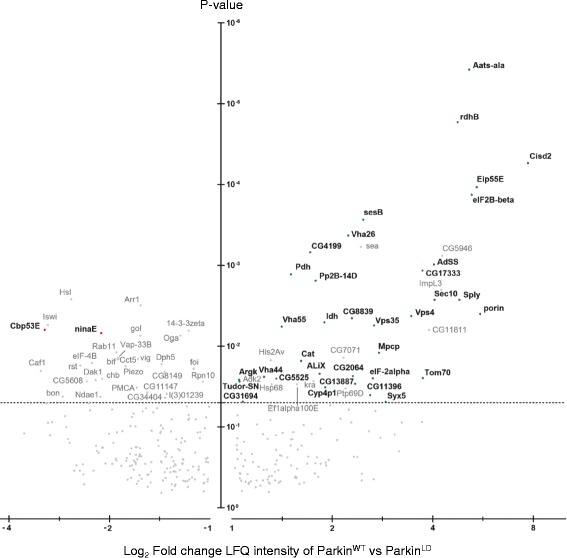
Distribution of ubiquitinated proteins according to Parkin catalytic activity. Volcano plot showing the outcome of the Parkin^WT^ vs Parkin^LD^ *T*-test performed with Perseus. Proteins are distributed along x axis by fold change of LFQ intensity and y axis by *p*-value. Proteins in bold, represent the most robust Parkin responders (35 enriched and 2 reduced - Table 1) whose LFQ change at the protein level is also coherently detected at the peptide level (Additional file 5: Figure S5)

Fifty-six proteins were discovered to be both more than 2-fold enriched and reach a significance of *p* < 0.05 (Perseus *T*-test, see Materials and Methods). In contrast, 30 proteins were reduced (using the same criteria) in purified ubiquitinated material from Parkin^WT^ over-expressing neurons compared to controls (Fig. 4, Additional file 6: Figure S6A). We next applied a further set of criteria to select the most robust Parkin responders (35 enriched, two reduced). These all provide at least two unique peptides in all three biological replicates and display a fold change bigger than two as average of all unique peptides (see Materials and Methods). Seven of the 35 enriched proteins are annotated as mitochondrial proteins, 12 as cytosolic and 13 as part of endomembrane system according to UNIPROT database (http://www.uniprot.org/) (Table 1, Additional file 10: Table S2). These include three sub-units of the v-ATPase required for endosome and lysosomal acidification, two proteins associated with the endosomal sorting complexes required for transport (ESCRT) machinery (ALiX, Vps4) and the PD-associated retromer component, Vps35. The membrane receptor NinaE (Opsin/Rhodopsin homolog) and the cytosolic calcium binding protein, Cbp53E are robustly, less ubiquitinated in a Parkin E3 ubiquitin ligase activity dependent manner.

**Table 1:**
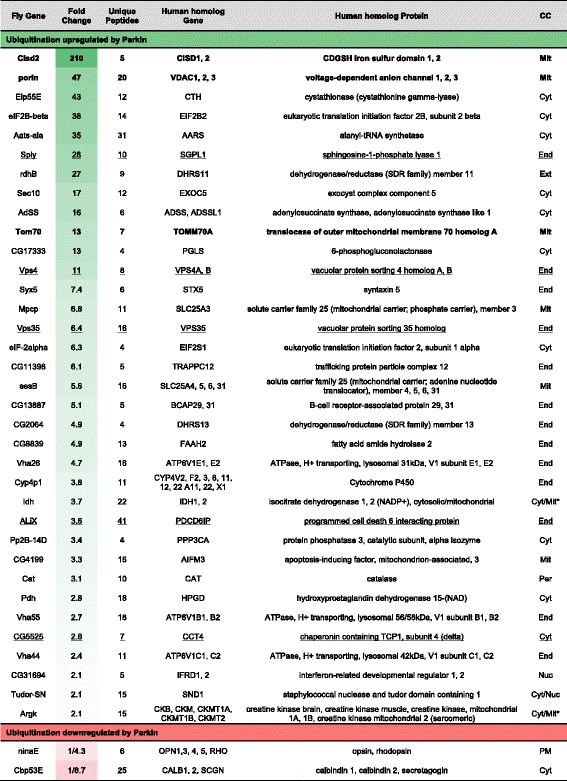
The most robust Parkin responders identified in *Drosophila* neurons

Proteins that are significantly more (green) and less (red) enriched in purified ubiquitinated material in a Parkin-dependent manner are shown in this table. Fold change refers to Parkin^WT^ vs Parkin^LD^ ratio in natural scale. Unique peptides found in the whole proteomic analysis are indicated. Human homolog genes were identified using g:Orth tool of web server g:Profiler. Cellular Compartment (CC) information was obtained from UniProt and is based on the human homolog. Cytosolic (Cyt), Mitochondrial (Mit), Endomembrane system (End), Extracellular (Ext), Peroxisomal (Per), Nuclear (Nuc) and Plasma Membrane (PM). Bold proteins represent known substrates and underlined proteins have been previously detected as more ubiquitinated in Parkin samples in previous Parkin proteomic studies in cell culture [37, 38]. *Cellular Compartment depends on the selected human homolog

**Fig. 5:**
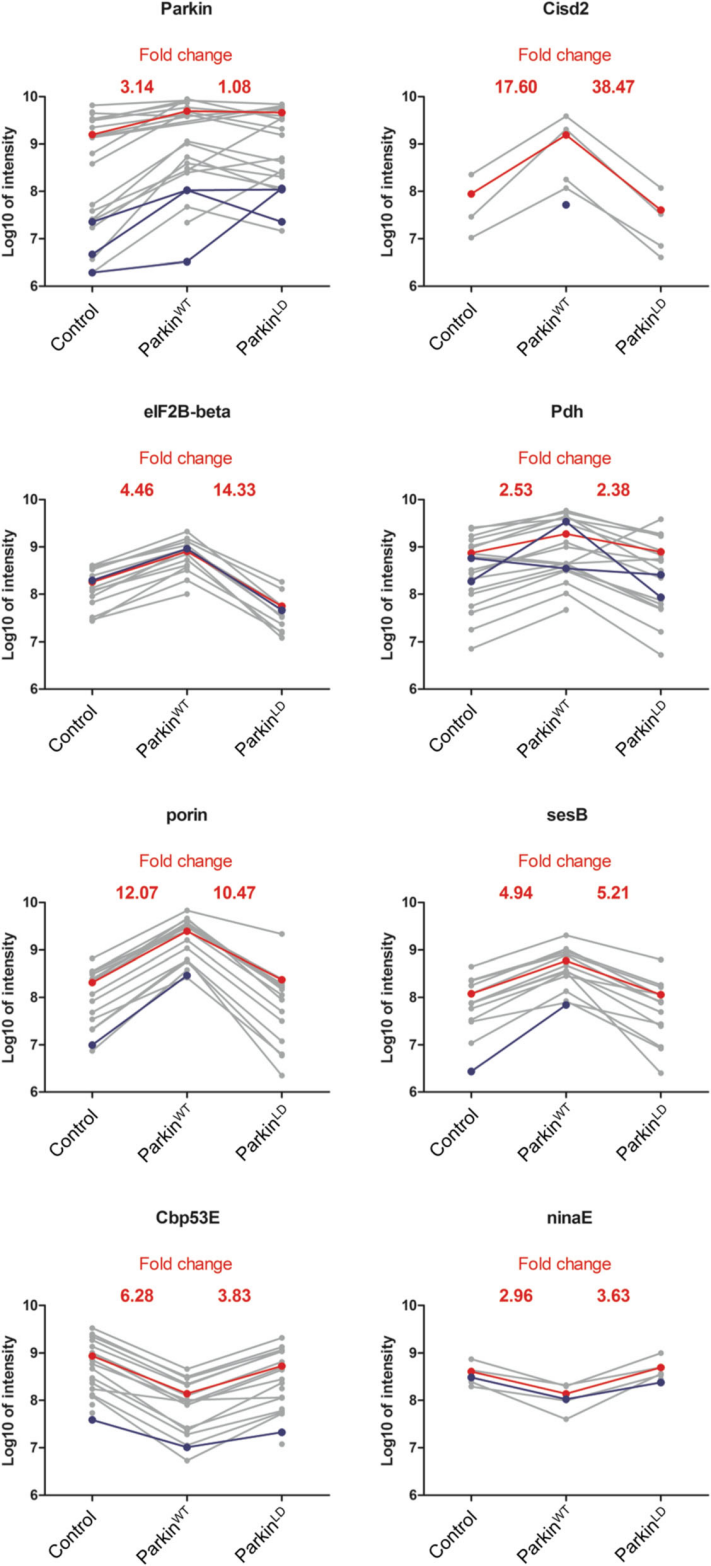
Peptide profile of robust Parkin responders encompassing a Di-Gly peptide. Di-Gly peptides were detected for 5 of the Parkin substrates identified: Cisd2, EIF2B-beta, Pdh, Porin and SesB. Moreover, Cbp53E and NinaE, whose ubiquitination robustly decreases in a Parkin E3 ubiquitin ligase activity dependent manner, also provided a di-Gly peptide. Three ubiquitination sites were also detected in Parkin itself. The graph represents the peptide intensity of all the unique peptides that were assigned to the indicated protein in each condition (*grey dots*). Di-Gly peptides are shown in *blue* and the average peptide intensity in *red*

Trypsin treatment of ubiquitinated proteins leaves a di-Gly signature residue at the ubiquitination site. Although the majority of peptides we use for quantitation are not themselves ubiquitinated, we have captured a set of 111 ubiquitinated peptides (Additional file 11: Table S3). Such di-Gly peptides were detected for five of the Parkin substrates identified as described above (Cisd2, EIF2B-beta, Pdh, Porin and SesB) and for the two proteins that were less ubiquitinated in a Parkin context (NinaE and Cbp53E) (Fig. 5). Di-Gly peptides corresponding to the following ubiquitination sites, Cisd2 K83, EIF2B-beta K30, Pdh K40, Porin K52 and SesB K120 were enriched in Parkin^WT^ over-expressing flies. However, Pdh K85 was found in a Parkin-independent manner, as similar intensities were detected in all samples. Di-Gly peptides for Cbp53E K262 and NinaE K372 were reduced in Parkin^WT^ flies. Moreover, 3 di-Gly peptides within Parkin itself were also detected that showed differing enrichment patterns. K35 was more ubiquitinated in Parkin^WT^ compared to both Parkin^LD^ and control. K40 was similarly ubiquitinated in Parkin^WT^ and Parkin^LD^, but not in control and K128 was much more ubiquitinated in Parkin^LD^ compared to Parkin^WT^ and control.

### Parkin preferentially generates K6 ubiquitin chains in Drosophila neurons

**Fig. 6:**
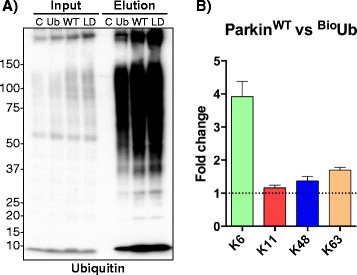
Parkin preferentially generates K6 ubiquitin chains. **a** C, Ub, WT, LD heads were subjected to ^Bio^Ub pulldown and inputs and elutions were immunoblotted for ubiquitin. **b** C, Ub, WT, LD fly heads were subjected to ^Bio^Ub pulldown and signature ubiquitin chain peptides were quantitated by comparison with isotopically labelled standards using mass spectrometry. Graph shows fold change of ubiquitin chain linkages found in Parkin^WT^ vs ^Bio^Ub. *Error bars* indicate standard deviation of three technical replicates. Complete genotypes of flies used: *GMR-GAL4/CyO;UAS-BirA/TM6* (C), *GMR-GAL4,UAS-(* ^*Bio*^ *Ub)*_*6*_ *-BirA/CyO* (Ub), *GMR-GAL4,UAS-(* ^*Bio*^ *Ub)*_*6*_ *-BirA/CyO;UAS-Parkin* ^*WT*^ (WT) and *GMR-GAL4,UAS-(* ^*Bio*^ *Ub)*_*6*_ *-BirA/CyO;UAS-Parkin* ^*LD*^ (LD)

Ubiquitin chains can be assembled through isopeptide bonds with any of the seven internal lysine residues. Each linkage type generates a unique peptide signature upon trypsin digestion that can be discriminated by mass spectrometry and quantitated by comparison with isotopically labelled standard [55]. We adopted this strategy to identify the linkage types of the ubiquitin chains that are generated by Parkin over-expression in vivo*,* by analysis of isolated ^Bio^Ub material. As judged by western blotting neither the overall pattern of ubiquitination nor the total amount of ubiquitinated material were altered by Parkin^WT^ over-expression in *Drosophila* neurons (Fig. 6a). The same conclusion can be obtained based on the Venn diagrams that compare the ubiquitomes identified upon Parkin over-expression with the control samples (Fig. 3b). However, we find a marked increase in the representation of K6 linked ubiquitin chains in Parkin^WT^ compared to control ^Bio^Ub and Parkin^LD^ samples (Fig. 6b, Additional file 6: Figure S6B).

### Validation of Parkin candidate substrates

**Fig. 7:**
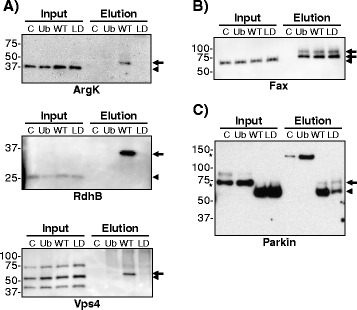
WB validation of in vivo Parkin substrates. C, Ub, WT, LD fly heads were subjected to ^Bio^Ub pulldown and Inputs and Elutions were immunoblotted for **a** RdhB, ArgK and Vps4; **b** Fax; **c** Parkin. WT and LD input and elution samples were diluted 50 and 25 times, respectively. *Arrowheads* show unmodified protein and arrows indicate ubiquitinated protein. *Asterisks* indicate unspecific bands. Complete genotypes of flies used: *GMR-GAL4/CyO;UAS-BirA/TM6* (C), *GMR-GAL4,UAS-(* ^*Bio*^ *Ub)*_*6*_ *-BirA/CyO* (Ub), *GMR-GAL4,UAS-(* ^*Bio*^ *Ub)*_*6*_ *-BirA/CyO;UAS-Parkin* ^*WT*^ (WT) and *GMR-GAL4,UAS-(* ^*Bio*^ *Ub)*_*6*_ *-BirA/CyO;UAS-Parkin* ^*LD*^ (LD)

We sought to validate ubiquitination of selected Parkin substrates by direct western blotting of proteins for which antibodies were available. For ArgK, RdhB and Vps4 we observe a fraction that is uniformly upshifted to a degree consistent with monoubiquitination in a manner that is stringently dependent on Parkin activity (Fig. 7a). In contrast, Fax, a protein that was not specifically enriched following Parkin expression in the mass spectrometry data set, is indiscriminately ubiquitinated in all samples (Fig. 7b). Parkin is enriched largely in an unmodified form in the eluates of flies over-expressing Parkin (Fig. 7c). This likely represents Parkin that is captured by virtue of a thioester-bonded ubiquitin at its active site cysteine, which is then released by the reducing conditions of the subsequent processing steps. The residual minor ubiquitinated fraction is linked via isopeptide bonds, suggesting that a small fraction of Parkin is itself ubiquitinated.

Parkin-dependent Vps35 ubiquitination could not be validated in flies by western blotting, due to the lack of available working antibodies for *Drosophila* Vps35. We instead performed in human cells an in vivo ubiquitination assay we recently developed [42, 43, 57, 58]. The main advantage of this method is that, due to the very stringent washes performed, the entire ubiquitin signal detected corresponds exclusively to the protein of interest and cannot be derived from other proteins in the cell. We tested this assay on SH-SY5Y neuroblastoma cells, which allowed us to further test whether Parkin-dependent ubiquitination of VPS35 is conserved in human cells.

**Fig. 8:**
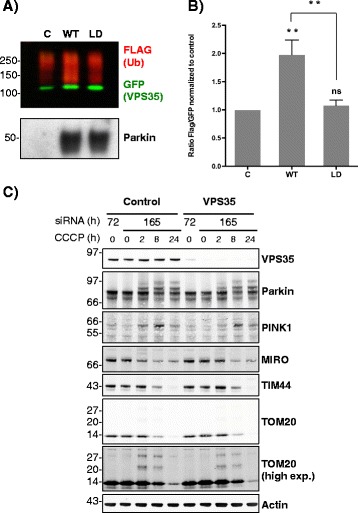
Human Parkin and VPS35 interaction: VPS35 is ubiquitinated by wild type Parkin in human cells. **a** VPS35 is ubiquitinated by untagged WT Parkin in SH-SY5Y cells. YFP-VPS35 showed a significant increase in its ubiquitinated fraction when it is over-expressed with wild type untagged hParkin (WT), as shown with anti-FLAG (to FLAG-Ub) antibody Western blot (*red*) compared to control (C) or the single point (C431S) mutated hParkin (LD). The non-modified form of VPS35 was detected by GFP antibody (*green*). The bottom panel shows levels of Parkin protein in the whole cell extract before the isolation of the GFP-tagged proteins. The endogenous Parkin protein is barely detected. **b** Quantification of the ubiquitination status of VPS35 relative to the non-modified form was performed calculating the ratio FLAG:GFP with Image-J. The plot shows relative levels of VPS35 ubiquitination normalized to the GFP levels. Statistical significance in Western blotting semi-quantification was evaluated using an analysis of variance (ANOVA) complemented by Tukey’s honest significance difference test (Tukey’s HSD) performed in GraphPad PRISM software. Statistical significance differences (**, *p* < 0.01 (mean ± SEM, *n* = 4)) were observed for the WT Parkin sample relative to both control and LD Parkin samples. For LD Parkin sample *ns* indicates not significant differences relative to the control sample. **c** VPS35 knockdown does not affect Parkin - dependent mitophagy. hTERT-RPE1 cells stably over-expressing YFP-Parkin were subjected to control and VPS35 siRNA for 72 h or 165 h and treated with 10 μM CCCP for 2, 8 and 24 h. Samples were immunoblotted as indicated. High exp: high exposure

SH-SY5Y cells were transfected with YFP-VPS35, FLAG-Ub and either WT or LD untagged human Parkin. Cells were then subjected to a GFP pulldown and the eluted samples monitored by Western blotting in order to compare ubiquitination levels of VPS35 across the different samples (Fig. 8a, Additional file 7: Figure S7). Since the ubiquitination signal proceeds only from YFP-VPS35, quantification of the data (Fig. 8b) is provided as ratios of the ubiquitin signal (monitored through the anti-Flag western) over the VPS35 intensity (monitored through the anti-GFP western). A significant increase in the ubiquitinated fraction of VPS35 was observed when WT Parkin was co-expressed, relative to either control (only endogenous Parkin or LD Parkin co-expression).

### VPS35 knockdown does not affect Parkin - dependent mitophagy in YFP-Parkin expressing RPE1 cells

Our results confirm a linkage between Parkin and the PD-associated protein VPS35, which has been previously revealed by genetic studies [59]. Both the proteomic data and the in vivo ubiquitination assay coupled to subsequent western blot validation suggest that VPS35 may be a substrate of Parkin, in line with previous studies that analysed Parkin substrates following acute mitochondrial depolarisation [38]. The mechanism that links VPS35 to PD is unclear, although recent evidence indicates that VPS35 regulates mitochondrial homeostasis by regulating fission-fusion dynamics [60, 61] and the trafficking of mitochondrial-derived vesicles (MDV) [62, 63]. We and others have recently used retinal pigment epithelial cells (RPE1) that stably over-express YFP-Parkin as a convenient system for analysis of Parkin-dependent mitophagy [32, 49]. We tested whether VPS35 is necessary for PINK1/Parkin - dependent mitophagy in these cells following acute mitochondrial depolarisation. However, siRNA-mediated knockdown of VPS35 had no effect upon mitophagy as judged by the time-dependent loss of several mitochondrial marker proteins (Fig. 8c).

## Discussion

### Parkin regulates a delicate balance between survival and cell death

Our finding that neuronal Parkin over-expression during neurodevelopment leads to multiple Parkinsonian-like defects conflicts with the simple notion that Parkin is neuroprotective [29]. However, recent cell culture and in vivo studies have shown that Parkin activity can also sensitize towards cell death in certain contexts [30–33]. We observed that the severity of Parkin over-expression phenotype is associated with its E3 ubiquitin ligase activity in a dosage dependent manner, which is further augmented during aging. Nevertheless, the fact that flies over-expressing Parkin^LD^ still have a mild phenotype, suggests that this mutant shows a dominant-negative effect that is not linked to the ligase function. Further in vivo experiments are necessary to unravel Parkin function at different developmental stages in different tissues*.*

### Functional roles of identified substrates

Identification of Parkin substrates is crucial in order to comprehend the still elusive aetiology of PD. While early studies stressed the notion that Parkin ubiquitinates and degrades several aggregation-prone toxic proteins, currently Parkin is mainly associated with mitophagy, through ubiquitination of several OMM proteins [35, 36, 64–66]. Parkin is predominantly held in an inactive conformation at steady state [20] and PINK1 dependent phosphorylation of both ubiquitin and Parkin is necessary for its activation [19–25]. Mitochondrial depolarisers have been proven to efficiently activate Parkin through PINK1 accumulation, but most cell lines show low levels of Parkin, precluding detectable levels of Parkin-dependent mitophagy and ubiquitination. Moreover, the ubiquitinated fraction of proteins is usually low due to their fast proteasomal degradation and/or the activity of deubiquitinating enzymes (DUBs) [67]. Therefore, most studies have been performed under conditions of Parkin over-expression using non-neuronal in vitro cell culture models, usually employing proteasome inhibitors and mitochondrial depolarisers. However, these acute conditions are not encountered in physiological settings. In the present study we have sought for in vivo Parkin substrates in *Drosophila* neurons, using a fly model that co-expresses constitutively biotinylated ubiquitin together with FLAG-tagged Parkin. While it has been shown that tagging human Parkin can lead to some constitutive activation [68], *Drosophila* Parkin has an extended N-terminal sequence relative to the human protein and addition of a further short sequence tag is not predicted to impact on the structure (Additional file 8: Figure S8). If such a scenario was extant, it would simply shift an equilibrium and potentially allow more sensitive detection of Parkin substrates without the recourse to acute drug treatments used in previous studies [27, 34, 36–38]. Treatments with mitochondrial depolarising drugs will lead to PINK1 accumulation and activation of Parkin specifically at mitochondria. Our study provides a complementary resource, which may capture some Parkin substrates that are PINK1-independent. The notion of alternative activation routes for Parkin has been previously proposed [69] and it is supported by data suggesting a role for Parkin in xenophagy that might be PINK1-independent [70].

**Fig. 9:**
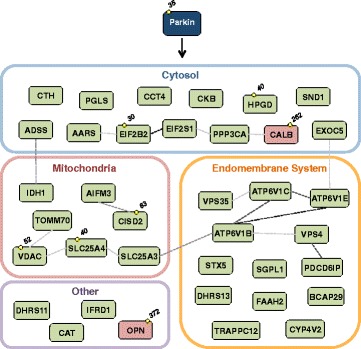
Functional connectivity of the most robust Parkin responders identified in *Drosophila* neurons. Cellular compartment representation of 35 enriched (*green*) and 2 reduced (*red*) human homolog proteins in purified ubiquitinated material from Parkin^WT^ over-expressing *Drosophila* neurons (Table 1). STRING (http://string-db.org/) analysis based on experimental data shows the connectivity. Strength of dotted lines represents differential significance (darker lines show higher significance). *Yellow circles* indicate Parkin^WT^ dependent ubiquitination sites identified in *Drosophila* proteins

We identified 37 proteins whose ubiquitination pattern changes in a Parkin E3 ubiquitin ligase activity dependent manner. 35 proteins were enriched and 2 were reduced in purified ubiquitinated material of Parkin over-expressing head extracts (Fig. 9). Western blot validation of proteins for which *Drosophila* antibodies were available confirmed several of these proteins as authentic in vivo Parkin substrates. A number of mitochondrial proteins were identified, including proteins known to be ubiquitinated during mitophagy, such as Cisd2/CISD1,2 (MitoNEET), Porin/VDAC1,2,3 and Tom70 [27, 34, 36–38, 71]. Nevertheless, in comparison to other recent Parkin proteomic studies, our Parkin substrate data set shows less enrichment of mitochondrial proteins, probably reflecting the lack of acute mitochondrial depolarization [27, 34, 37, 38]. Whilst some of the mitochondrial proteins are ultimately matrix associated, they are synthesised in the cytosol and imported through the TIM/TOM complex providing the opportunity to encounter cytosolic Parkin. Interestingly, the presence of ubiquitinated proteins within the yeast mitochondrial matrix has recently been reported [72]. In fact, a recent paper suggested that Parkin ubiquitinates and regulates the stability of the mitochondrial matrix enzyme 17-β hydroxysteroid dehydrogenase type 10 (HSD17B10) [73]. Further proteins previously detected also include Sply/SGPL1, VPS4, VPS35, ALiX/PDCD6IP and CG5525/CCT4 [37, 38]. To our knowledge, the remaining 27 enriched proteins are novel Parkin substrates, which are predominately cytosolic and endosomal proteins. Inversely, NinaE/Opsin, Rhodopsin and Cbp53E/Calbindin were reduced in purified ubiquitinated material after Parkin over-expression indicating that Parkin can indirectly regulate the ubiquitination status of these proteins. It is also plausible that Parkin-dependent ubiquitination followed by rapid degradation result in reduced protein levels leading to decreased ubiquitination status of these substrates. Importantly, decreased calbindin mRNA and protein levels are linked to neurodegeneration in PD [74–77].

The fact that there is no singular pathway controlled by substrates described here suggests that under in vivo physiological conditions Parkin may have broader roles beyond the control of mitophagy. For example, we note prominent representation of endosomal-associated proteins, including three sub-units of the v-ATPase required for endosome and lysosomal acidification, two proteins associated with the endosomal sorting complexes required for transport (ESCRT) machinery (ALiX/PDCD6IP, VPS4) and the PD-associated gene and retromer component VPS35. Moreover, proteins involved in protein synthesis, metabolic process, cell death, calcium signaling and immune response are also present (Additional file 10: Table S2). Further study of Parkin substrates will reveal whether they act in an unexplored common pathway.

### Parkin monoubiquitinates substrates and preferentially generates K6 ubiquitin chains

A single ubiquitin can be attached to a unique (monoubiquitination) or several (multiple-monoubiquitination) lysine residues of substrate proteins. Ubiquitin can be generated from these stubs to form chains of different topologies (M-1, K6, K11, K27, K29, K33, K48, K63). This provides a complex “ubiquitin code” that regulates a myriad of biological processes. Whilst K48 regulates protein turnover, through proteasomal degradation, and K63 is mainly involved in endosomal trafficking and DNA repair, the function of the “non-canonical” chains is less well defined [78, 79]. Parkin has been reported to be able to promote monoubiquitination as well as multiple chain types such as K6, K11, K27, K48 and K63 ubiquitin chains in diverse contexts through interaction with different E2s [64, 80–83]. Moreover, Parkin has been shown to autoubiquitinate itself via K6 ubiquitin chains and the deubiquitinase USP8 has been suggested to remove these K6 chains to regulate Parkin stability and its recruitment to mitochondria [84]. Furthermore, USP15, USP30 and USP35 have been shown to deubiquitinate Parkin substrates and/or regulate mitophagy following acute mitochondrial depolarisation in cells [32, 38, 85, 86]. While USP15 has been reported to have a preference for K48 and K63 [85], USP30 has been reported to favour K6 chains disassembly [87].

In contrast to our earlier work using this same bioUb pulldown and Western blotting approach [39, 40, 42, 43], most of the specific proteins we have validated here by western blotting were found to be monoubiquitinated. This agrees with previous observations demonstrating that Parkin is able to generate monoubiquitinated conjugates [81, 82]. Our ubiquitin chain analysis showed that Parkin preferentially promotes K6 chain formation in contrast to some literature reports indicating that Parkin promotes a more diverse set (K6, K11, K48 and K63) after mitochondrial depolarisation [83, 88]. However, in line with our observations, recent ubiquitin chain analysis showed that K6 is preferentially formed in whole lysates from Parkin over-expressing HEK and SH-SY5Y cells after mitochondrial depolarisation [87].

### Implications of Parkin-dependent VPS35 ubiquitination in neurodegeneration

Despite the fact that the exact molecular mechanism leading to PD is unclear, the set of genes linked to PD can be divided in two main pathways. Parkin, DJ-1, PINK1 and FBXO7 are linked to mitochondrial homeostasis [65, 66, 89, 90], while α-synuclein (SNCA), LRRK2, VPS35, EIF4G1 and ATP13A2 are involved in endosomal-lysosomal trafficking [7–9]. LRRK2, RAB7L1 and VPS35 have been shown to act in a common pathway [91, 92] and VPS35 has recently been shown to genetically interact with EIF4G1 in a pathway upstream of α-synuclein [93]. Furthermore, VPS35 impairment leads to lysosomal dysfunction and α-synuclein pathology [94, 95].

The finding that Parkin ubiquitinates VPS35 is of considerable interest, as it could represent a connection between mitochondrial homeostasis and endo-lysosomal pathways, unifying the PD-associated genes in a common network [96]. In fact, recent studies in *Drosophila* reported that *Vps35* genetically interacts with *parkin* [59]. Our identification of VPS35 as a Parkin substrate strengthens this association. Moreover, VPS35 has been found to be involved in mitochondrial homeostasis [60, 61], therefore it is tempting to consider that Parkin-dependent ubiquitination of VPS35 may regulate mitophagy. However, our results show that Parkin-dependent mitophagy is not affected by VPS35 knockdown, suggesting that VPS35 is not an essential regulator of Parkin-dependent mitophagy in a mammalian cell system subjected to acute depolarisation. Nor do we see any changes in VPS35 levels following Parkin activation (Fig. 8c) or with control RPE-1 cells which do not express Parkin (not shown). Alternatively, Parkin - dependent-ubiquitination of VPS35 could be involved in the regulation of mitochondrial-derived vesicles (MDV), which have been reported to traffic defective mitochondrial proteins to peroxisomes and lysosomes in a more specific manner prior to the whole organelle impairment [62, 63, 97]. A further possibility is that Parkin ubiquitinates and regulates endo-lysosomal pathway components through the ubiquitination of several proteins, including VPS35 and others described here. During the preparation of this manuscript Parkin has been reported to modulate endo-lysosomal trafficking through Rab7 ubiquitination, but also to influence the distribution of VPS35 and the levels of the retromer cargo protein ci-M6PR [98]. Other recent papers have also linked Parkin to endo-lysosomal trafficking [99–102]. The involvement of endocytic trafficking in PD is gaining momentum with the finding that two main PD-associated genes, PINK1 and LRRK2, converge and lead to the phosphorylation of common Rab proteins, small GTPases involved in membrane trafficking [103–105].

Interestingly, both VPS35 and Parkin mutations are also associated with Alzheimer’s Disease (AD) [106–108]. VPS35 interacts with the amyloid precursor protein (APP) sorting receptor, SorL1, which is also an AD causing gene, to regulate APP processing and Aβ production [109–113]. Parkin has recently been identified as an AD risk gene [108] and decreased Parkin levels have been found in AD patient cerebrospinal fluids [114]. Remarkably, Parkin over-expression has been shown to ameliorate AD symptoms by increasing the clearance of amyloid loads [115, 116], which may suggest that defects in a common endo-lysosomal pathway are shared between PD and AD. In this scenario, Parkin may have a role in endosomal trafficking control through ubiquitination of VPS35, with a potential impact on both PD and AD. Certainly, further work is required in order to explore this hypothesis.

## Conclusion

Constitutive expression of biotinylated ubiquitin has been proven to be a successful approach to study ubiquitination in vivo [39, 40, 43]. Here we extend this method to identify Parkin substrates in adult *Drosophila* neurons. We report 35 substrates, eight of which have been previously detected and 27 are novel. Notably we identified VPS35 as an in vivo Parkin substrate, both in flies and in human neuroblastoma SH-SY5Y cells, reinforcing a connection between mitochondrial homeostasis and endo-lysosomal pathways in PD and AD.

## Additional files

Additional file 1: Figure S1. Parkin over-expression in developing neurons causes severe climbing defects. A) Anti-Parkin and Syntaxin1A western blot of stated genotypes. Head extracts of flies over-expressing two copies of Parkin (Parkin^WT^ and Parkin^LD^) were diluted 4 times. B) Whole climbing assay shown in Fig. 1d. *Stars* indicate 0% climbing ability and a skull represents complete death at the particular time point. C) GFP, Flag-Parkin^WT^, Flag-Parkin^LD^, untagged Parkin^WT^ R1, Parkin^WT^ R2, 8HA-Parkin WT/+ (one copy) and 8HA-Parkin WT (two copies) constructs were expressed with *elav-Gal4*. Climbing assay of stated genotypes was performed as well as western blot for Parkin and Syx1A. Parkin^WT^ R1, Parkin^WT^ R2 are *elav-Gal4* recombined versions of *parkin* from [16]. Three technical replicates were performed with three biological replicates of each genotype and age. Columns represent average values and error bars show standard error of the mean (SEM). Statistical significance was assessed by two-tailed paired Student’s-t test. *Asterisk*(s) indicate significance, *p ≤ 0.05, **p ≤ 0.01 and ***p ≤ 0.001. Complete genotypes of flies used: *elav-GAL4,UAS-GFP/CyO* (GFP), *elav-GAL4/CyO;UAS-Parkin* ^*WT*^ */TM6* (Parkin^WT^/+), *elav-GAL4/CyO;UAS-Parkin* ^*LD*^ */TM6* (Parkin^LD^/+), *elav-GAL4/CyO;UAS-Parkin* ^*WT*^ (Parkin^WT^), *elav-GAL4/CyO;UAS-Parkin* ^*LD*^ (Parkin^LD^) *and park* ^*25*^ (Parkin null), *elav-GAL4,UAS-Parkin/CyO* (Parkin R1, R2), *elav-GAL4/CyO;UAS-8HAParkin* ^*WT*^ */TM6* (8HA-Parkin/+) and *elav-GAL4/CyO;UAS-8HAParkin* ^*WT*^ (8HA-Parkin). (PDF 268 kb)

Additional file 2: Figure S2. Control western blots A) The complete gel of Fig. 2f showing the three independent ^Bio^Ub pulldown experiments analysed by MS for Parkin substrate identification. B) All individual loading controls (actin western blots) for Fig. 8. (PDF 337 kb)

Additional file 3: Figure S3. Reproducibility of the system I. A) Venn diagrams show number of proteins detected in each ^Bio^Ub pulldown analysed by MS for Parkin substrate identification. B) LFQ intensity distribution of identified proteins in all ^Bio^Ub pulldowns used for Parkin substrate identification with Perseus software. Non-inputated values are shown in green and inputated values in red. Note that Perseus replaced LFQ intensities that displayed a value of 0 by low value intensities within the lower detection limit according to a normal distribution. LFQ intensities are displayed in Log_2_ scale. (PDF 785 kb)

Additional file 4: Figure S4. Reproducibility of the system II. Multicorrelation graph of LQF intensities of proteins identified in all ^Bio^Ub pulldowns analysed by MS for Parkin substrate identification. LFQ intensities are displayed in Log_2_ scale. (PDF 149 kb)

Additional file 5: Figure S5. Peptide validation to identify the most robust Parkin responders. According to Perseus *T*-Test (Fold change >2, *p*-value < 0.05), 51 and 11 proteins were significantly more or less enriched respectively in purified ubiquitinated material of Parkin over-expressing head extracts, in a Parkin-dependent manner at the protein level with at least 2 unique peptides in all three relevant conditions (for enriched Parkin^WT^ and for reduced Parkin^LD^). To further identify which are the ones that are more robustly associated to Parkin activity, the peptide profile of each of those proteins was analysed (see material and methods). A) Intensities of all the unique peptides in each condition (grey dots) and the average peptide intensity (red) are represented for each protein. When present, di-gly peptides are showed in blue. B) Fold change of all the unique peptides in each condition (grey dots) and the average fold change and standard error of the mean (SEM) are represented in red. Average intensity of the three replicates was used per condition and only peptides that appeared at least in two conditions were used. (PDF 3020 kb)

Additional file 6: Figure S6. Proteins that are specifically more ubiquitinated after Parkin expression in two independent analyses and supplementary Ub chain graph. A) Outcome of two T-tests performed with Perseus are plotted in this volcano plot: Parkin^WT^ vs Parkin^LD^ (left side) and Parkin^WT^ vs Control (^Bio^Ub; right side). Proteins are distributed along x axis by fold change of LFQ intensity and y axis by *p*-value. Proteins with a fold change bigger than 2 and a *p*-value lower than 0.05 are labelled with their names in grey. Proteins in bold, represent the most robust Parkin responders (see Table 1, Additional file 5: Figure S5). Fold change of LFQ intensity is represented in Log2 scale. B) C, Ub, WT, LD fly heads were subjected to ^Bio^Ub pulldown and ubiquitin chains present in elutions were quantitated by comparison with isotopically labelled standards. Graph shows fold change of ubiquitin chain linkages found n Parkin^WT^ vs Parkin^LD^. Error bars indicate standard deviation of three technical replicates. Complete genotypes of flies used: *GMR-GAL4/CyO;UAS-BirA/TM6* (C), *GMR-GAL4,UAS-(* ^*Bio*^ *Ub)*_*6*_ *-BirA/CyO* (Ub), *GMR-GAL4,UAS-(* ^*Bio*^ *Ub)*_*6*_ *-BirA/CyO;UAS-Parkin* ^*WT*^ (WT) and *GMR-GAL4,UAS-(* ^*Bio*^ *Ub)*_*6*_ *-BirA/CyO;UAS-Parkin* ^*LD*^ (LD). (PDF 414 kb)

Additional file 7: Figure S7. Over-expression of hParkin (WT) induced an increase of VPS35 ubiquitination compared to control (C) or to over-expression of inactive hParkin (LD) in four independent experiments. (A) The complete gels of Fig. 8a showing four independent in vivo ubiquitination assays for VPS35 in SH-SY5Y cells. Ubiquitination of YFP-tagged VPS35 was analysed by Western blot after capture of the YFP-tagged protein. Mouse anti-GFP antibody was used for detecting the captured VPS35 (shown in green), and HRP-conjugated anti-FLAG antibody for monitoring its ubiquitinated fraction (shown in red). Untagged Parkin over-expression levels in the whole cell extracts are monitored with anti-Parkin antibody. (B) Quantification of the ubiquitination status of VPS35 relative to the non-modified form was performed calculating the ratio FLAG:GFP with Image-J. The plot shows relative levels of VPS35 ubiquitination normalized to the GFP levels. (PDF 14700 kb)

Additional file 8: Figure S8. Alignment of Human and *Drosophila* Parkin. (PDF 61 kb)

Additional file 9: Table S1. All proteins identified: Details of all proteins identified in the whole mass spectrometry analysis. Proteins are divided in Background and Hits (Ubiquitinated). (XLS 2430 kb)

Additional file 10: Table S2. The most robust Parkin responders identified in *Drosophila* neurons: Details of the most robust Parkin responders identified in *Drosophila* neurons. (XLS 58 kb)

Additional file 11: Table S3. Di-gly Sites: Details of all detected Di-gly peptides. (XLS 154 kb)

Additional file 12: Video S1. Parkin over-expression in neurodevelopment results in Parkinsonian-like defects: Empty vials containing 5 male and 5 female, 0–3 days after eclosion flies of *elav-GAL4/CyO;UAS-Parkin* ^WT^ in the left side and *elav-GAL4/CyO;UAS-Parkin* ^LD^ in the right side. (M4V 4550 kb)

## Abbreviations

AD: Alzheimer’s Disease
APP: Amyloid precursor protein
DUBs: Deubiquitinating enzymes
ESCRT: Endosomal sorting complexes required for transport
LFQ: Label Free Quantification
MDV: Mitochondrial-derived vesicles
MS: Mass Spectrometry
OMM: Outer Mitochondrial Membrane
PD: Parkinson’s Disease
PINK1: PTEN-induced kinase 1
RBR: RING between RING
RPE1: Retinal pigment epithelial cells
Ub: Biotinylated ubiquitin/^Bio^Ubiquitin
UBL domain: Parkin Ubiquitin-like
