# Supplementary figures and images for: Quantitative proteomic analysis of Parkin substrates in Drosophila neurons
Source: Mol Neurodegener. 2017 Apr 11;12:29. doi: 10.1186/s13024-017-0170-3 (PMC5387213; doi:10.1186/s13024-017-0170-3)

A)

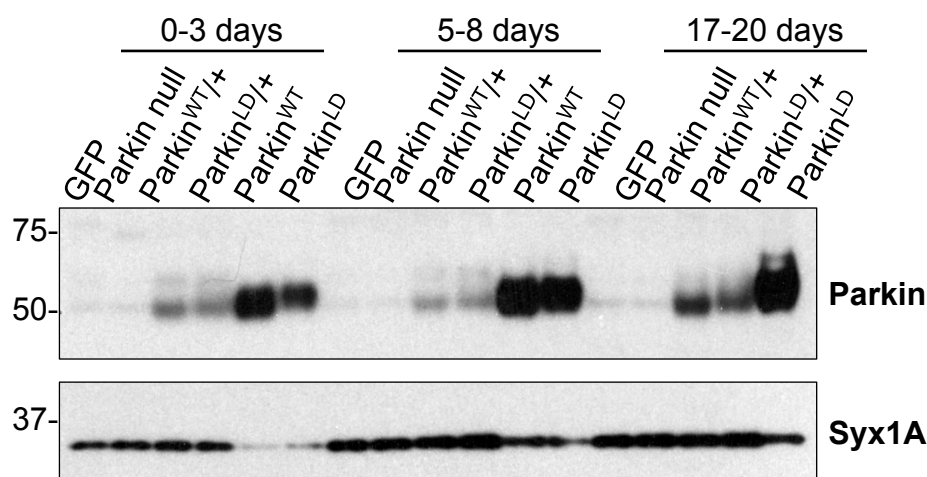

B)

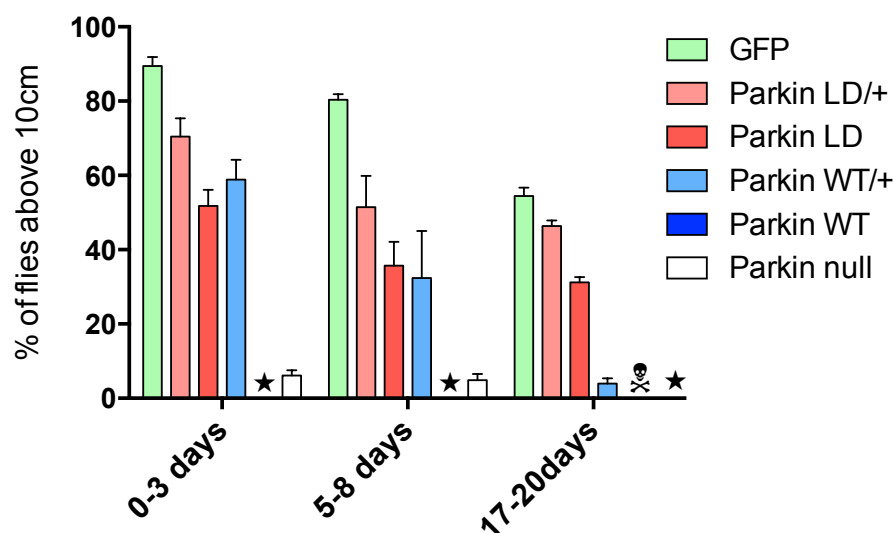

C)

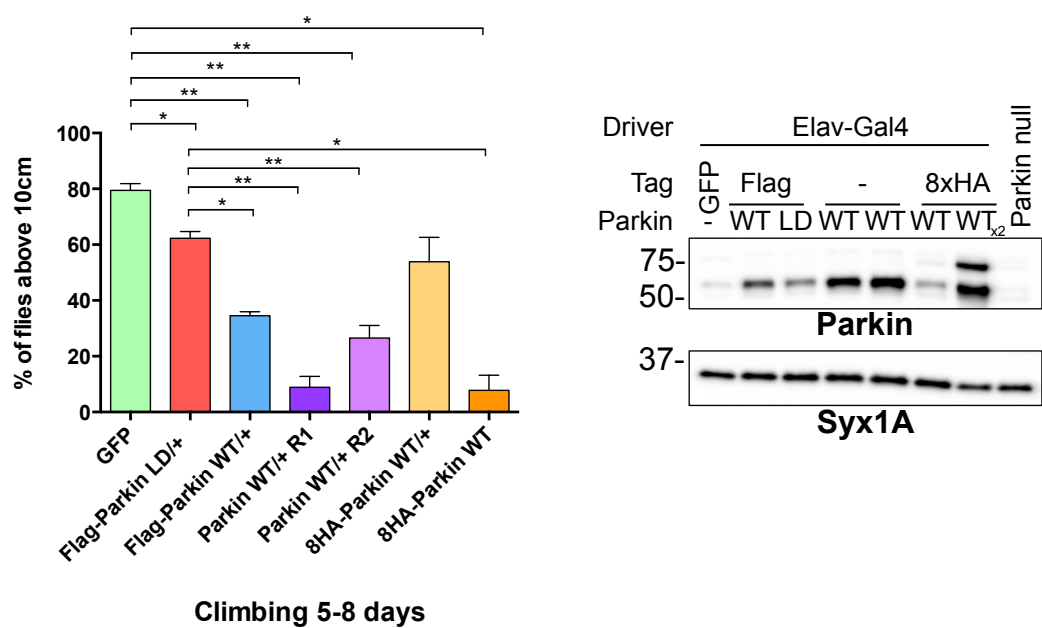

Supplementary Figure 1

Supplement: Supplementary file 1 — Parkin over-expression in developing neurons causes severe climbing defects. A) Anti-Parkin and Syntaxin1A western blot of stated genotypes. Head extracts of flies over-expressing two copies of Parkin (ParkinWT and ParkinLD) were diluted 4 times. B) Whole climbing assay shown in Fig. 1d. Stars indicate 0% climbing ability and a skull represents complete death at the particular time point. C) GFP, Flag-ParkinWT, Flag-ParkinLD, untagged ParkinWT R1, ParkinWT R2, 8HA-Parkin WT/+ (one copy) and 8HA-Parkin WT (two copies) constructs were expressed with elav-Gal4. Climbing assay of stated genotypes was performed as well as western blot for Parkin and Syx1A. ParkinWT R1, ParkinWT R2 are elav-Gal4 recombined versions of parkin from [16]. Three technical replicates were performed with three biological replicates of each genotype and age. Columns represent average values and error bars show standard error of the mean (SEM). Statistical significance was assessed by two-tailed paired Student’s-t test. Asterisk(s) indicate significance, *p ≤ 0.05, **p ≤ 0.01 and ***p ≤ 0.001. Complete genotypes of flies used: elav-GAL4,UAS-GFP/CyO (GFP), elav-GAL4/CyO;UAS-Parkin WT /TM6 (ParkinWT/+), elav-GAL4/CyO;UAS-Parkin LD /TM6 (ParkinLD/+), elav-GAL4/CyO;UAS-Parkin WT (ParkinWT), elav-GAL4/CyO;UAS-Parkin LD (ParkinLD) and park 25 (Parkin null), elav-GAL4,UAS-Parkin/CyO (Parkin R1, R2), elav-GAL4/CyO;UAS-8HAParkin WT /TM6 (8HA-Parkin/+) and elav-GAL4/CyO;UAS-8HAParkin WT (8HA-Parkin). (PDF 268 kb) [file 13024_2017_170_MOESM1_ESM.pdf]

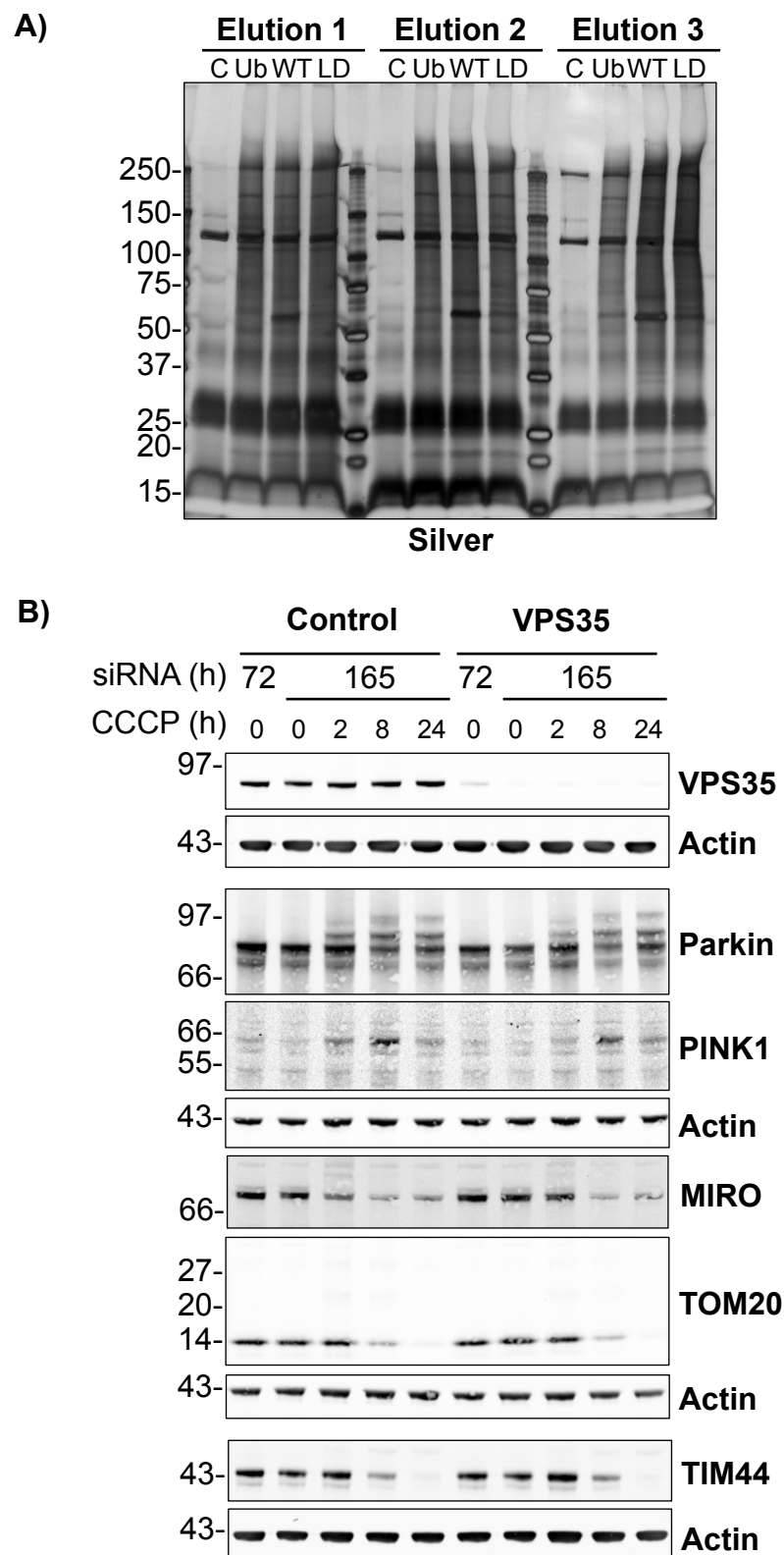

Supplementary Figure 2

Supplement: Supplementary file 2 — Control western blots A) The complete gel of Fig. 2f showing the three independent BioUb pulldown experiments analysed by MS for Parkin substrate identification. B) All individual loading controls (actin western blots) for Fig. 8. (PDF 337 kb) [file 13024_2017_170_MOESM2_ESM.pdf]

A)

No. of proteins  
detected in

EXP1

EXP2

EXP3

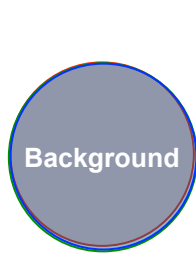

380  
388  
385

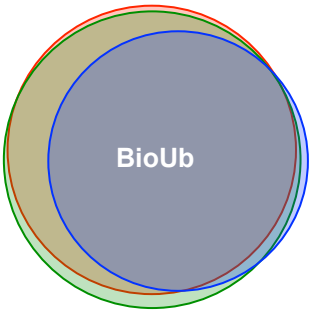

971  
1026  
775

Total 390

Total 1056

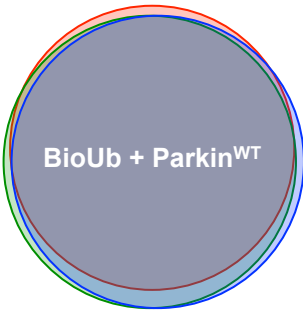

987  
1013  
1011

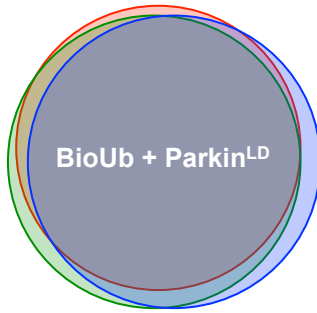

978  
998  
1010

Total 1077

Total 1096

B)

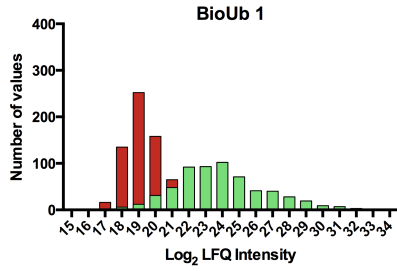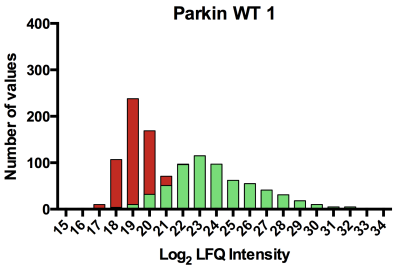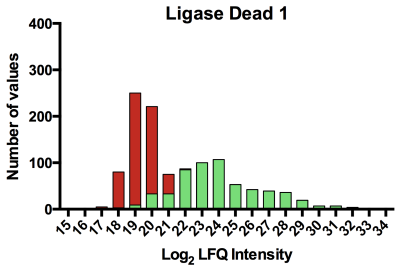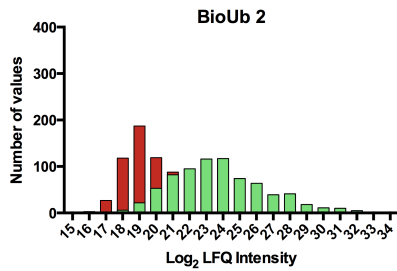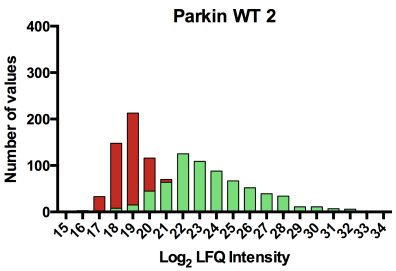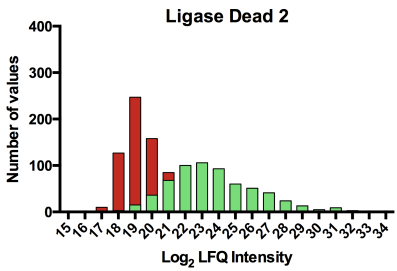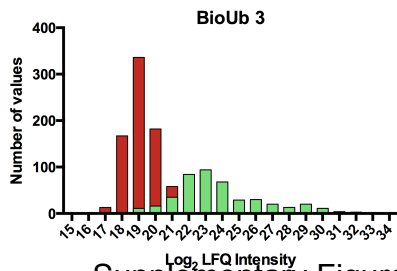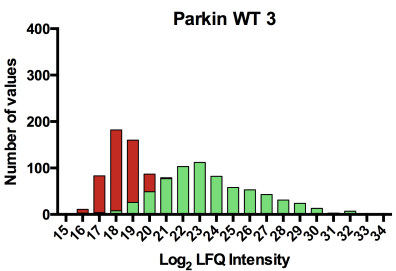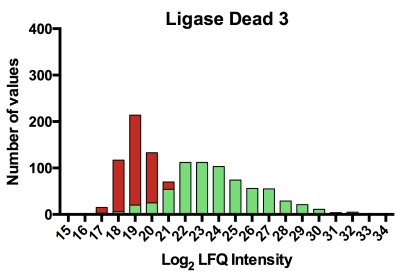

Supplementary Figure 3

Supplement: Supplementary file 3 — Reproducibility of the system I. A) Venn diagrams show number of proteins detected in each BioUb pulldown analysed by MS for Parkin substrate identification. B) LFQ intensity distribution of identified proteins in all BioUb pulldowns used for Parkin substrate identification with Perseus software. Non-inputated values are shown in green and inputated values in red. Note that Perseus replaced LFQ intensities that displayed a value of 0 by low value intensities within the lower detection limit according to a normal distribution. LFQ intensities are displayed in Log2 scale. (PDF 785 kb) [file 13024_2017_170_MOESM3_ESM.pdf]

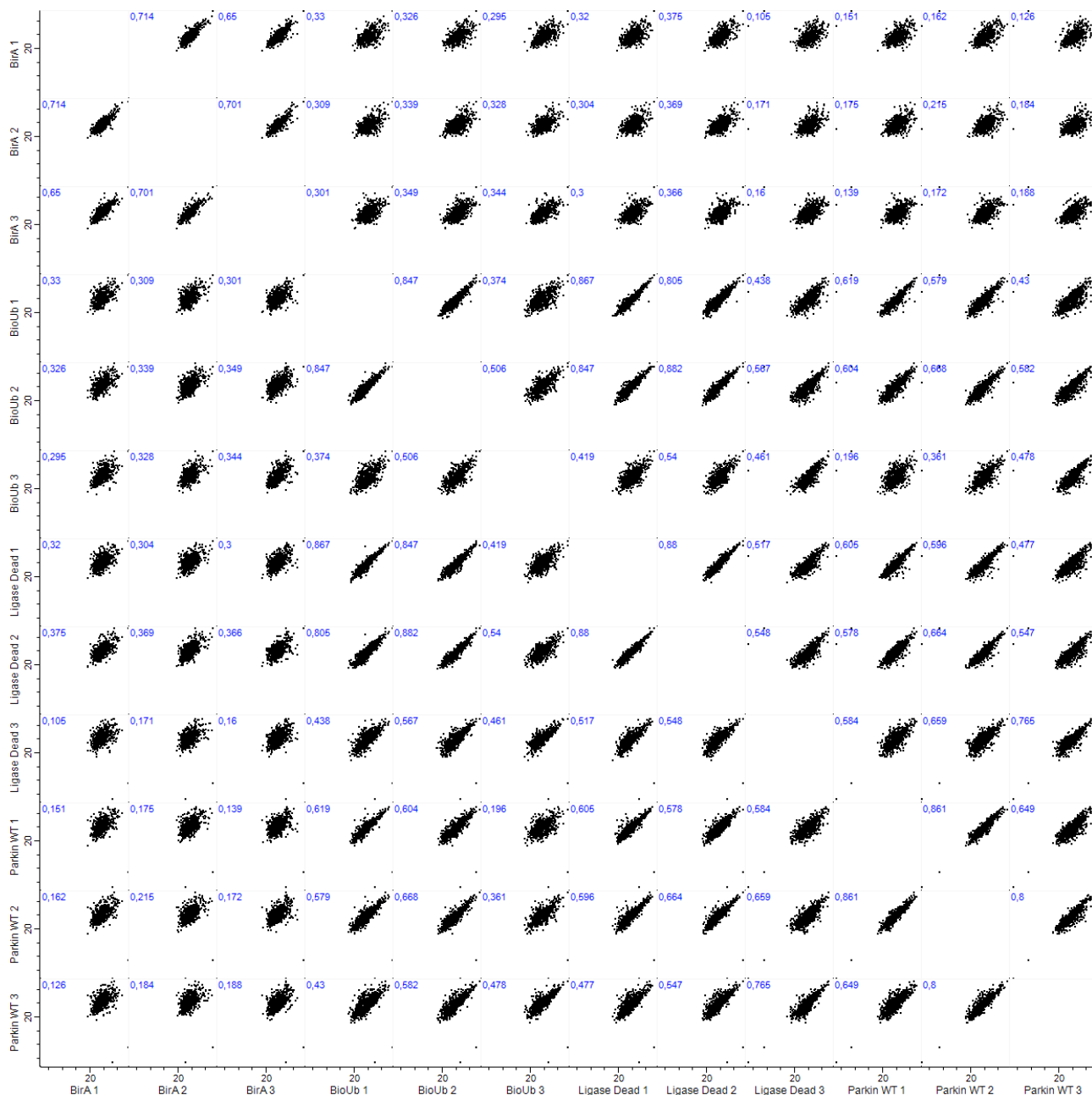

Supplementary Figure 4

Supplement: Supplementary file 4 — Reproducibility of the system II. Multicorrelation graph of LQF intensities of proteins identified in all BioUb pulldowns analysed by MS for Parkin substrate identification. LFQ intensities are displayed in Log2 scale. (PDF 149 kb) [file 13024_2017_170_MOESM4_ESM.pdf]

A)

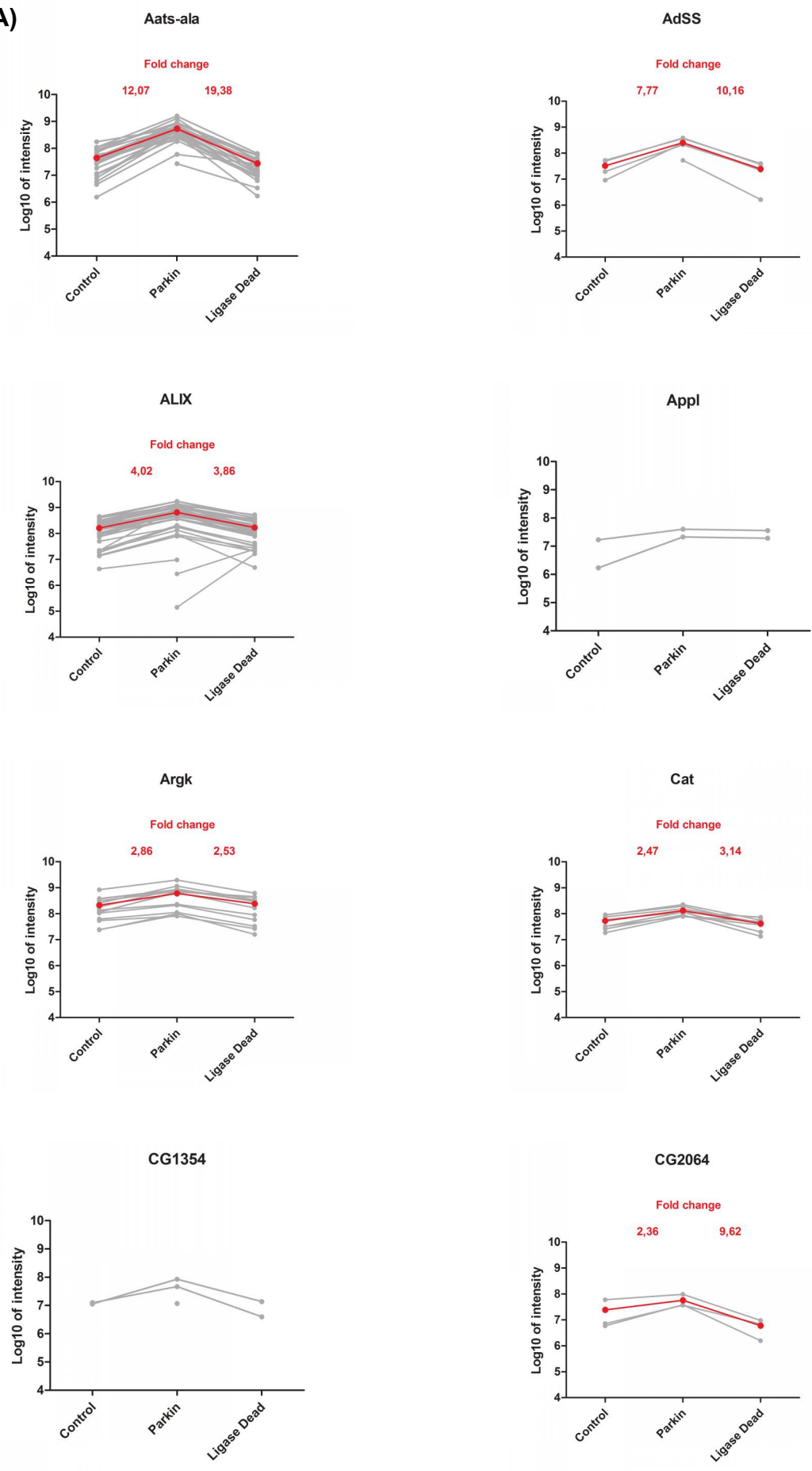

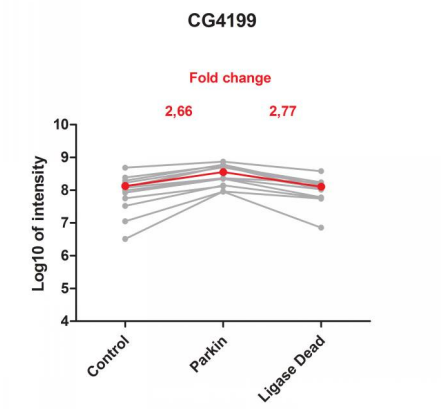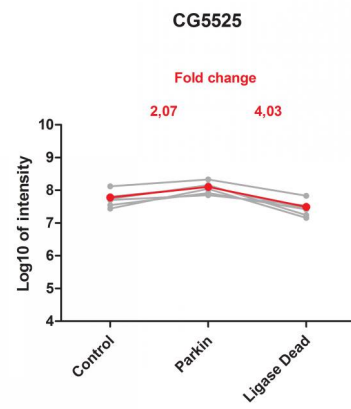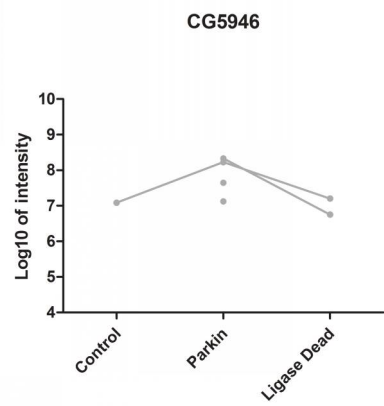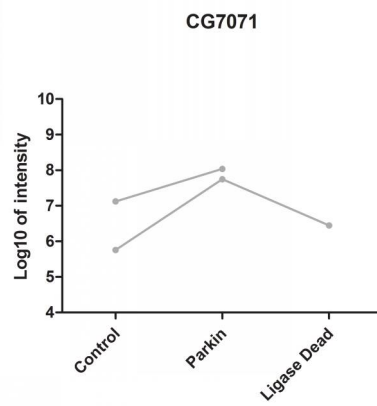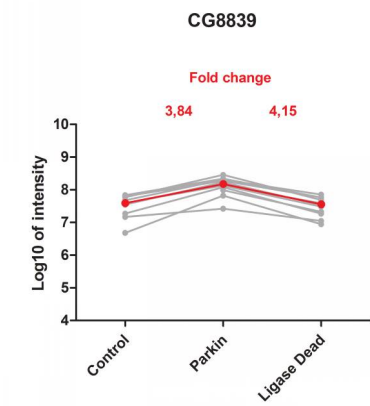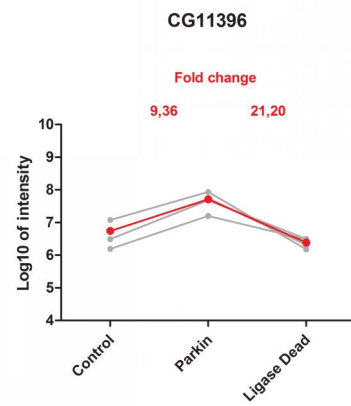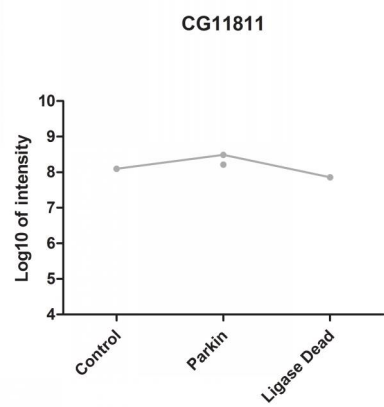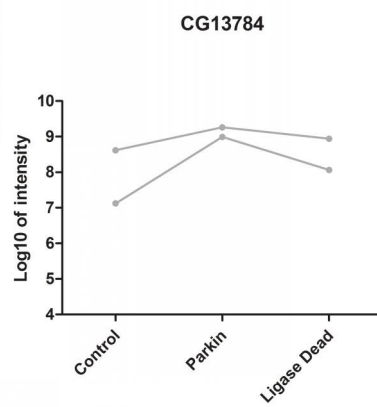

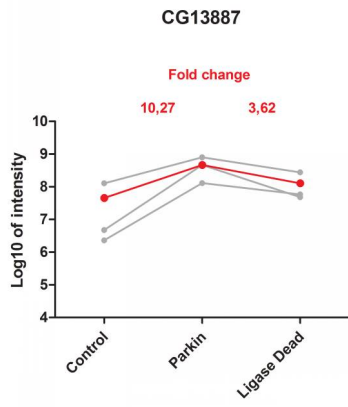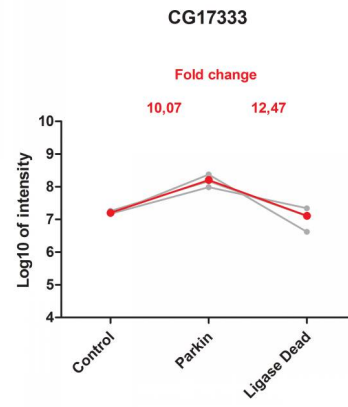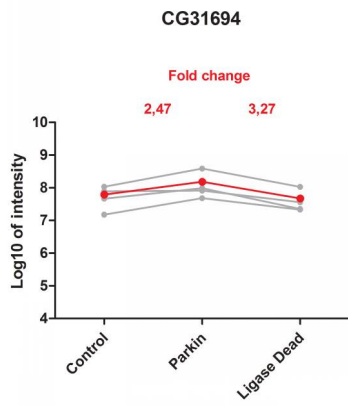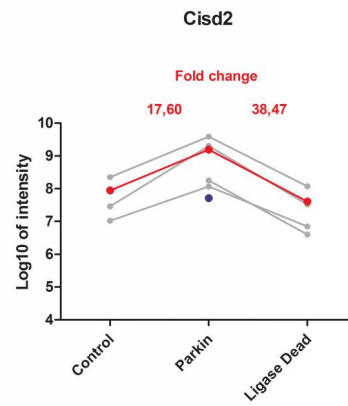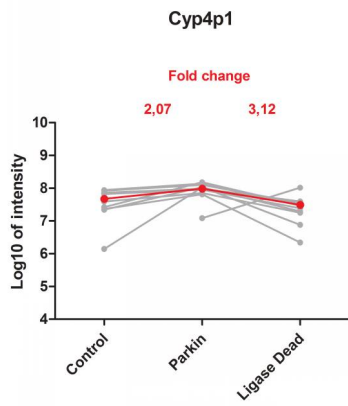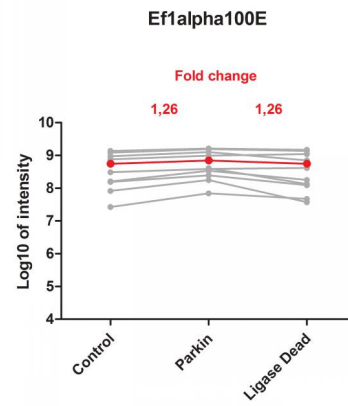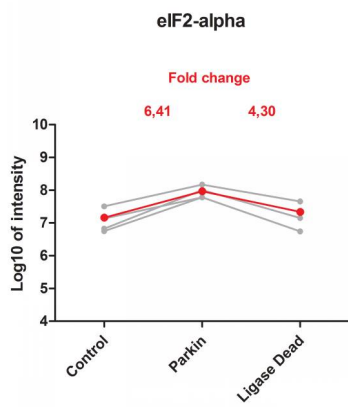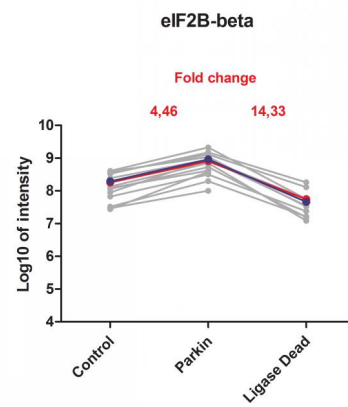

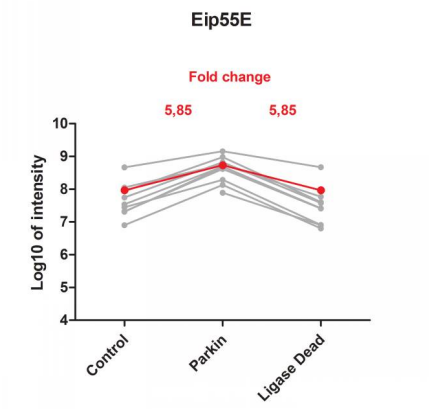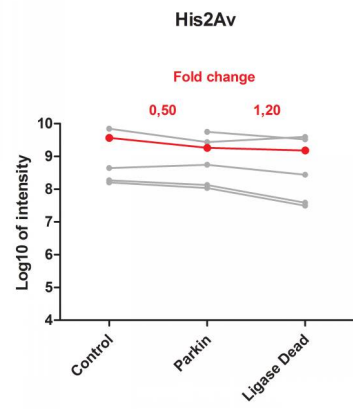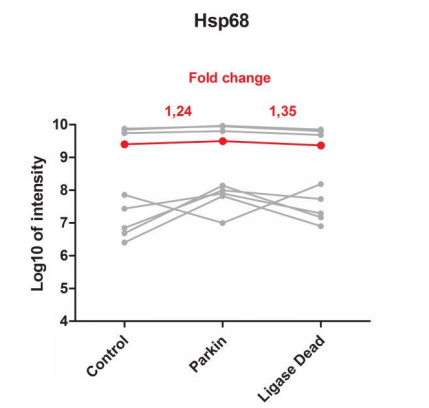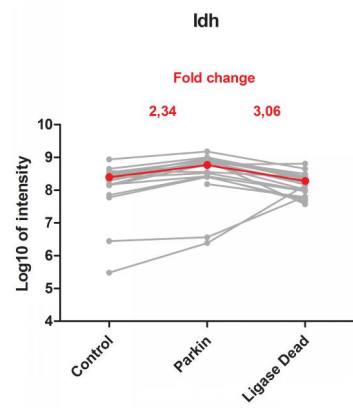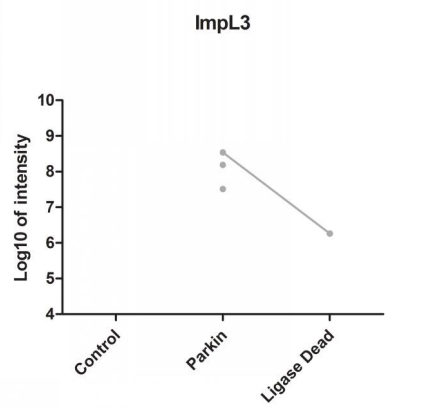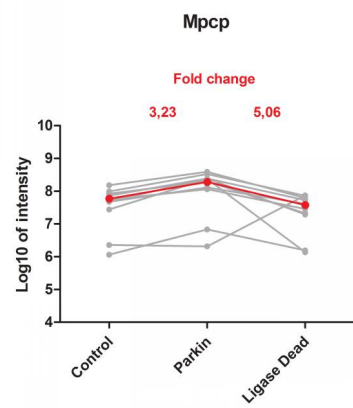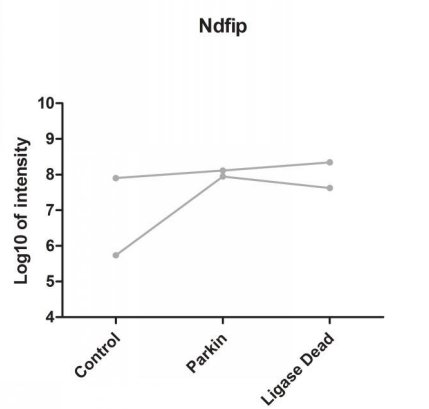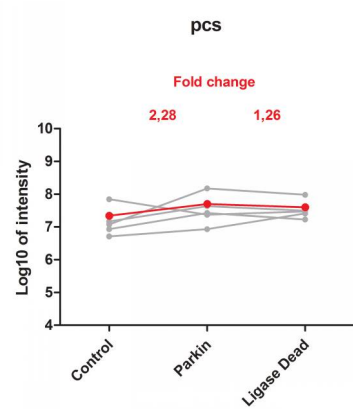

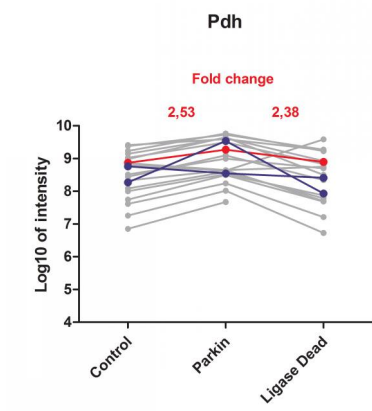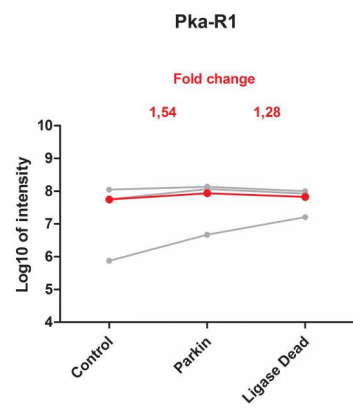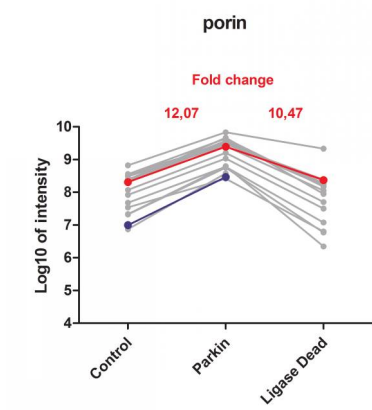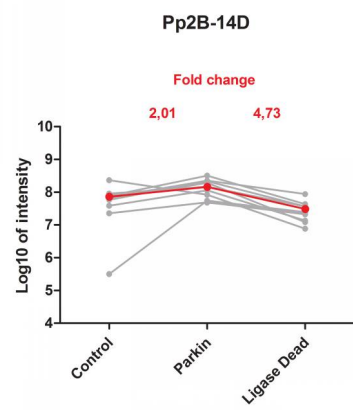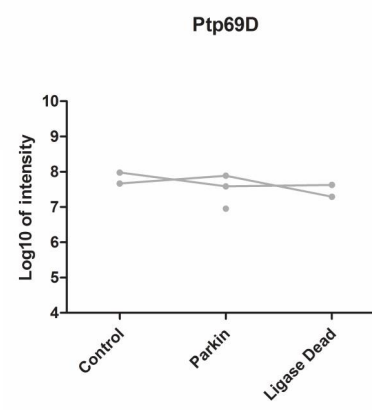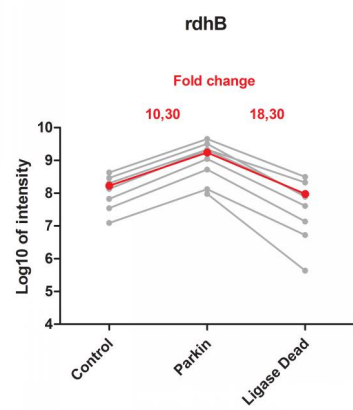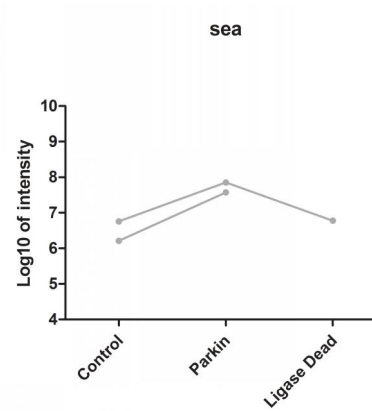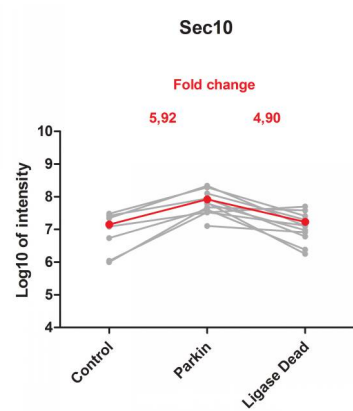

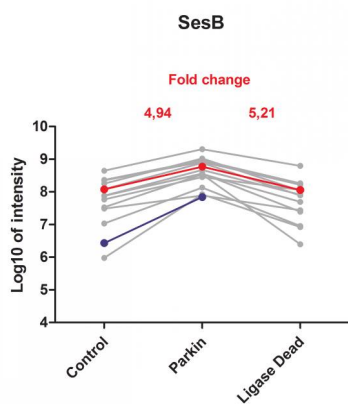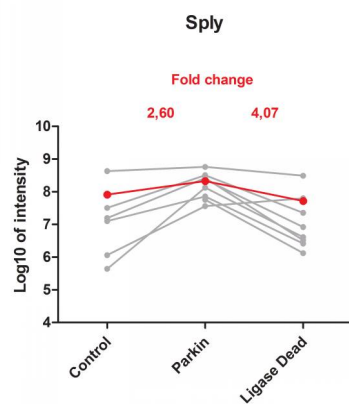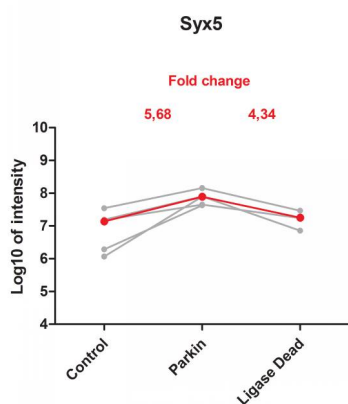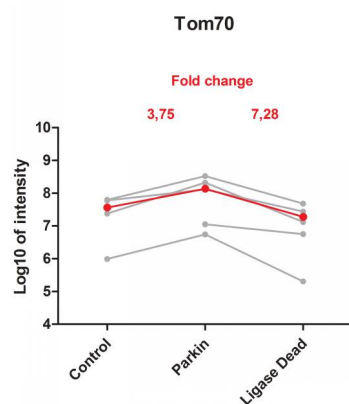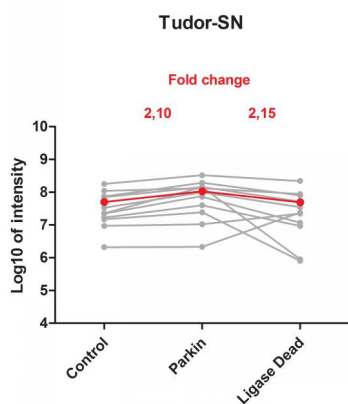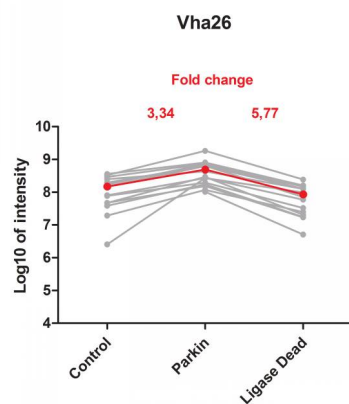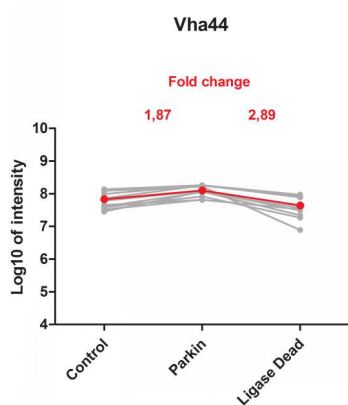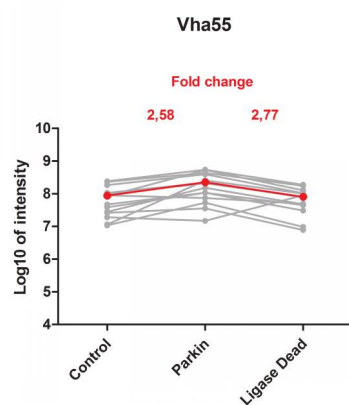

Vha68-1

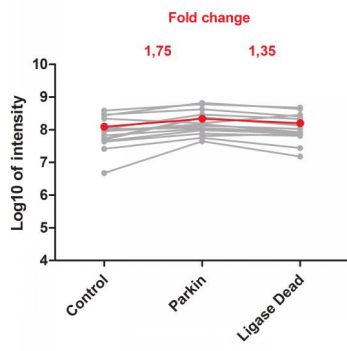

Vps4

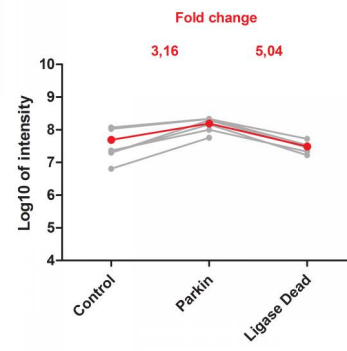

Vps35

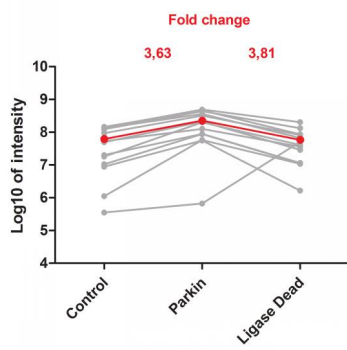

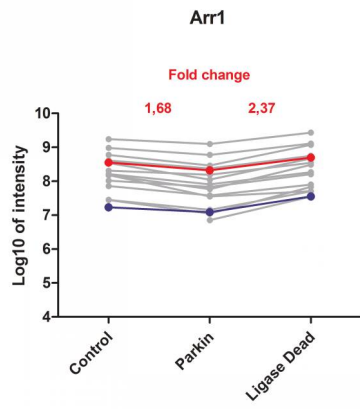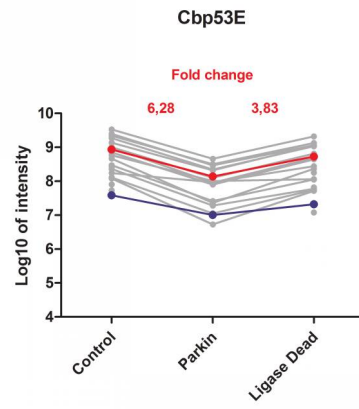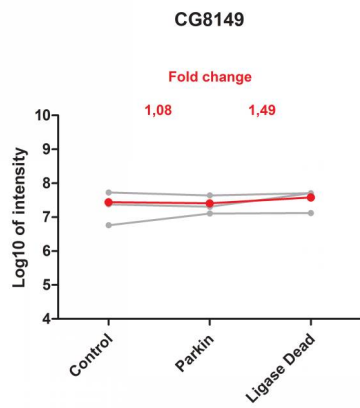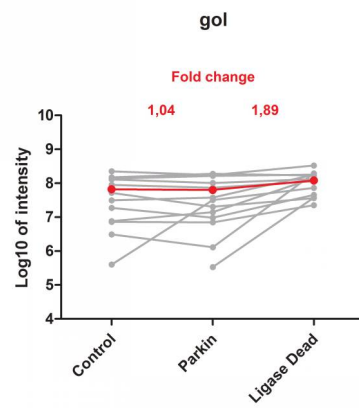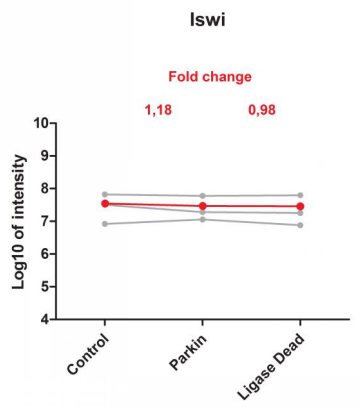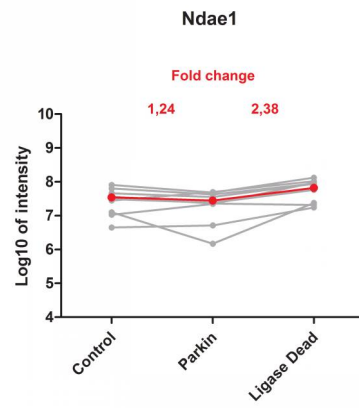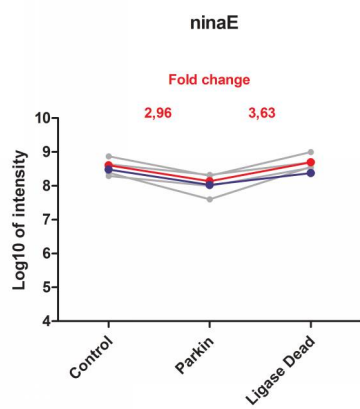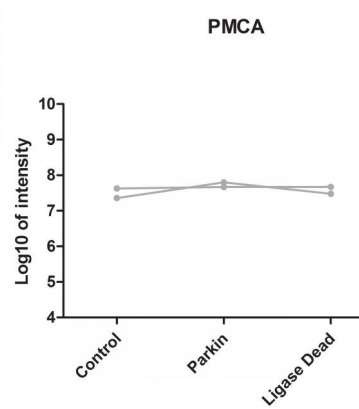

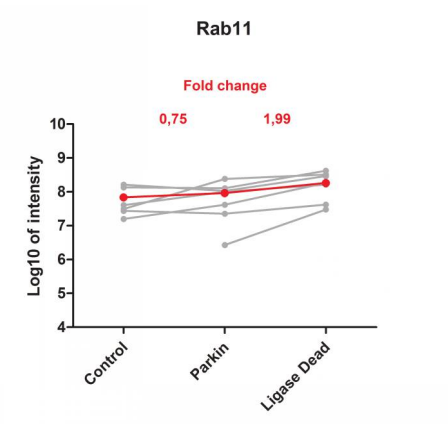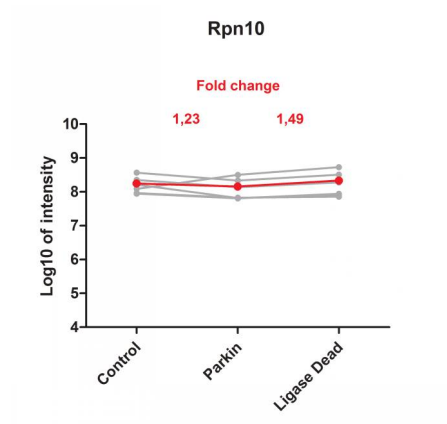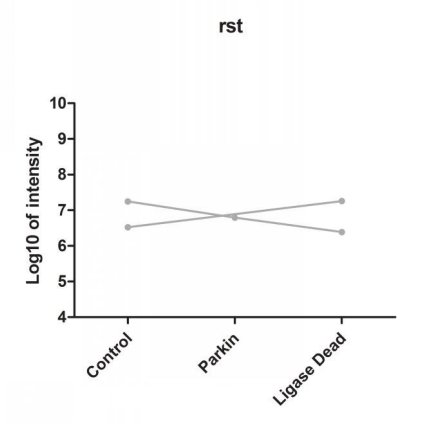

B)

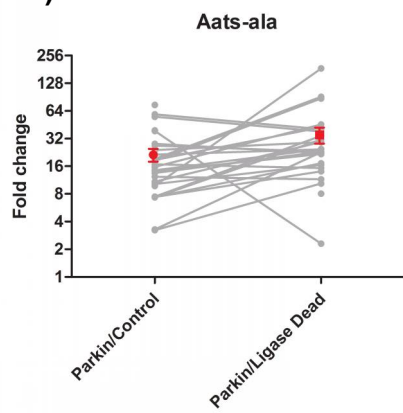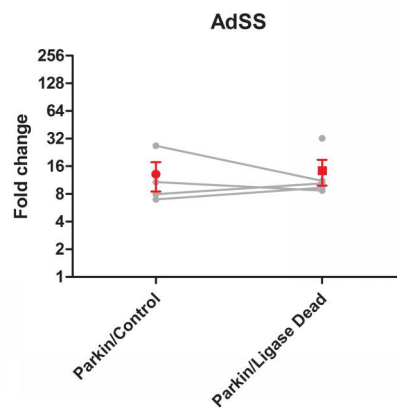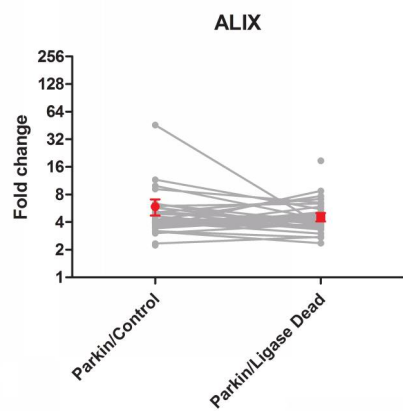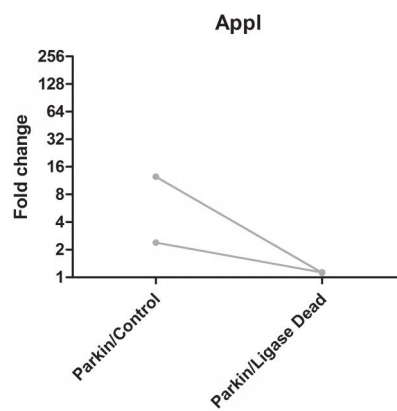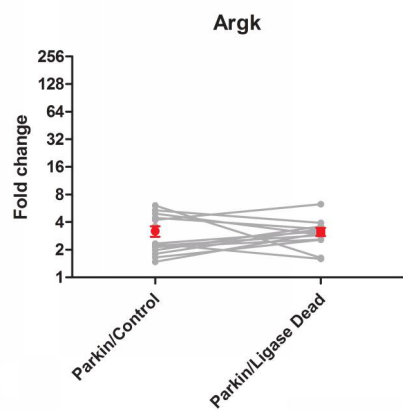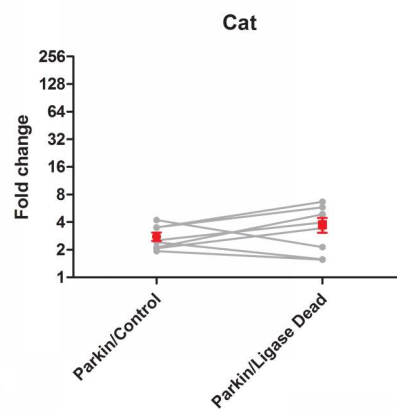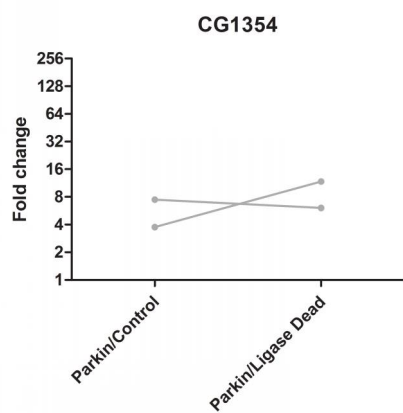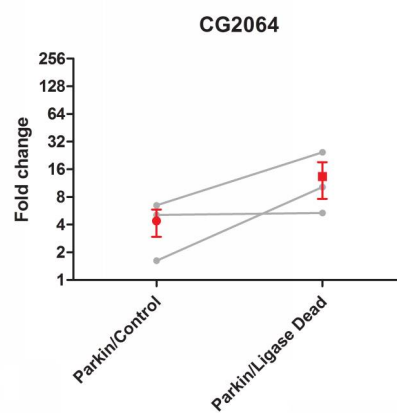

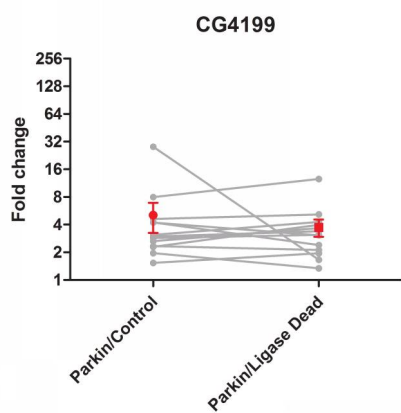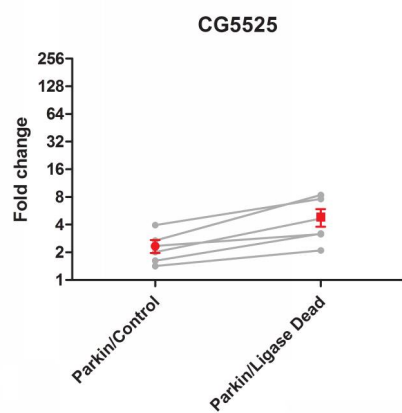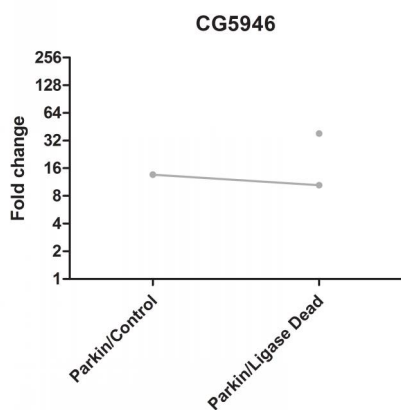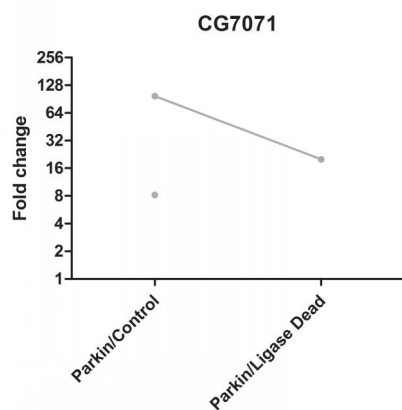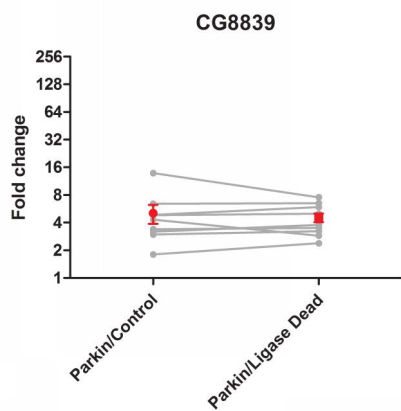

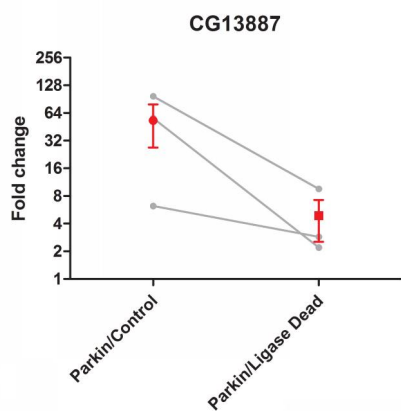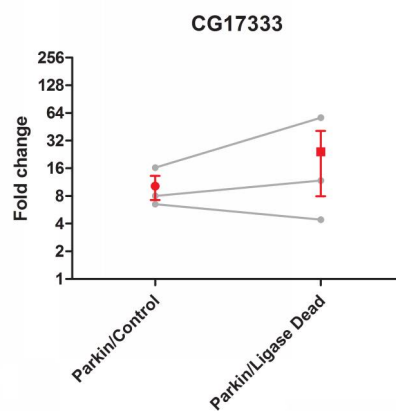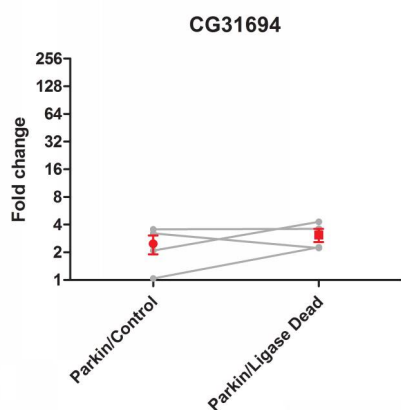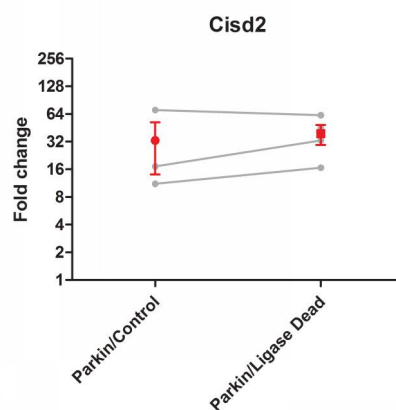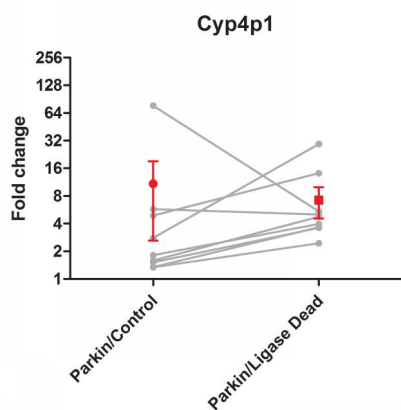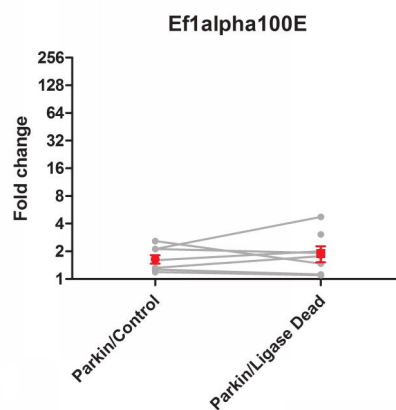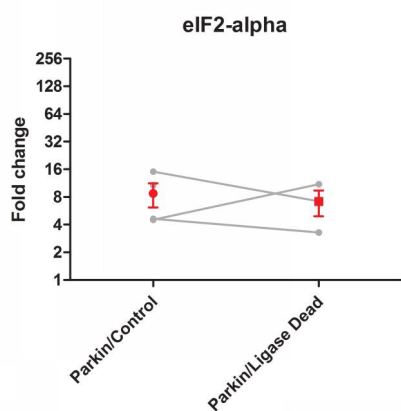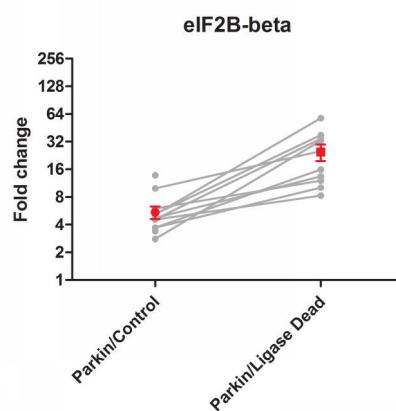

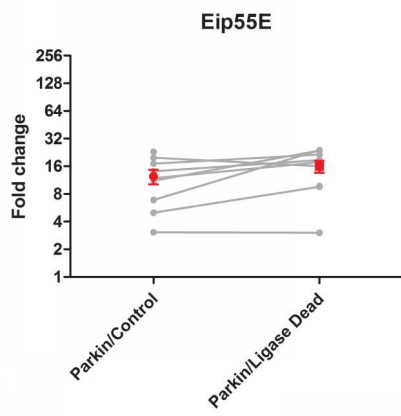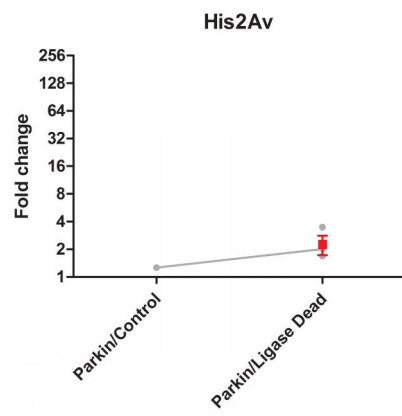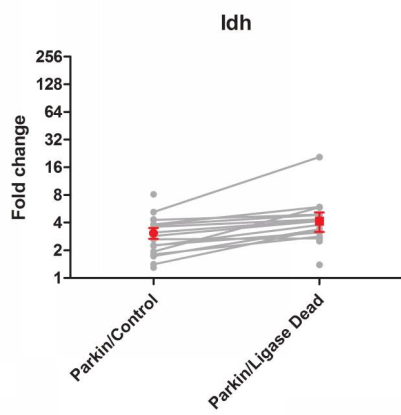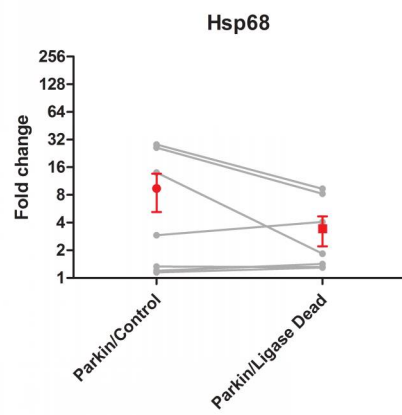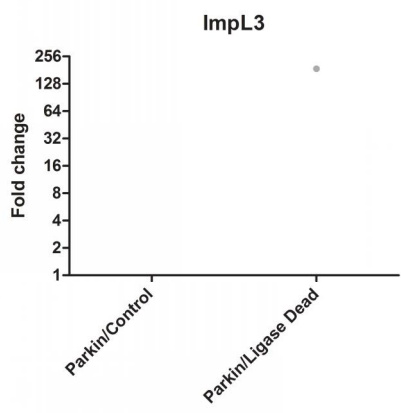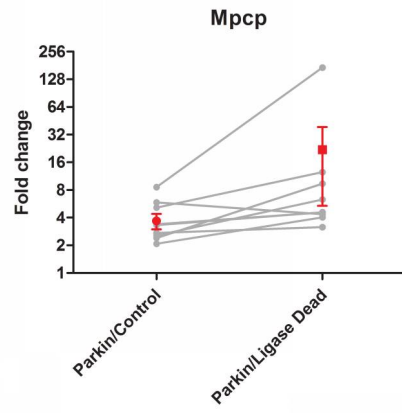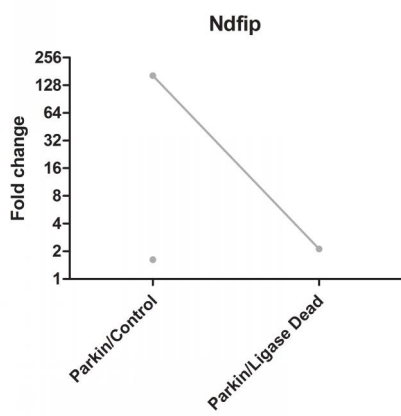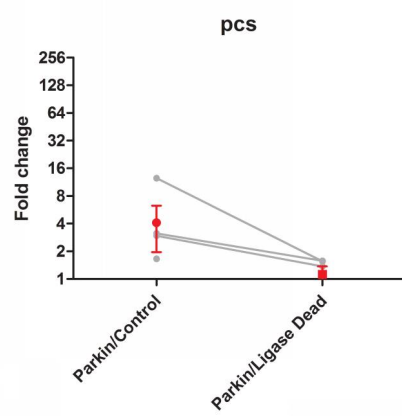

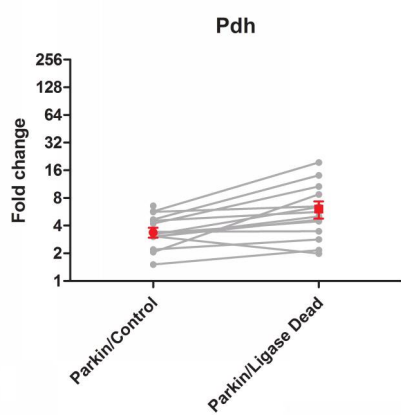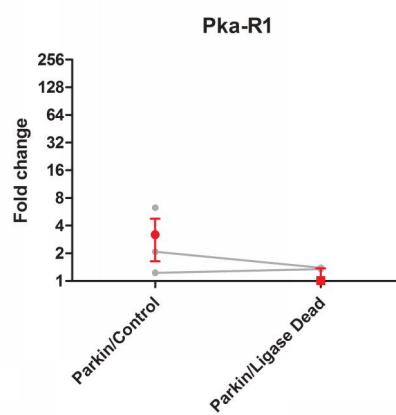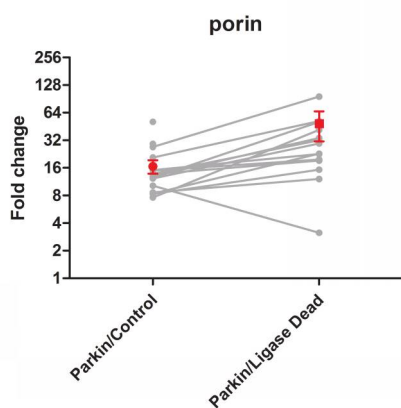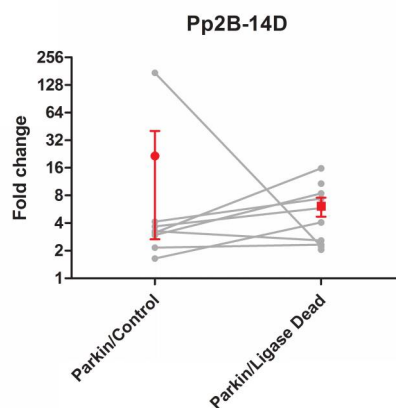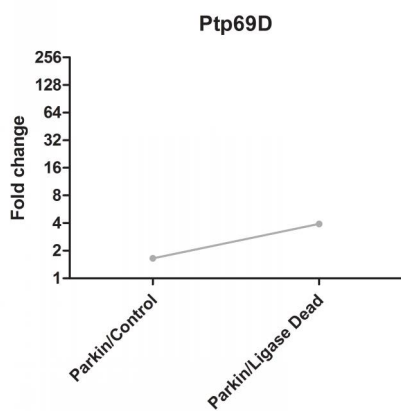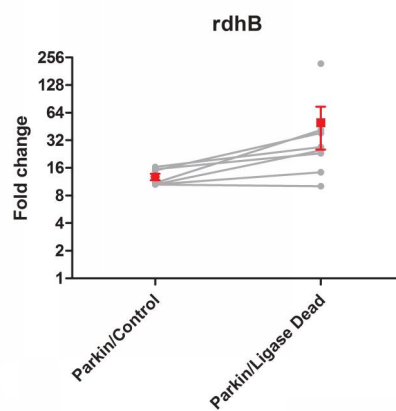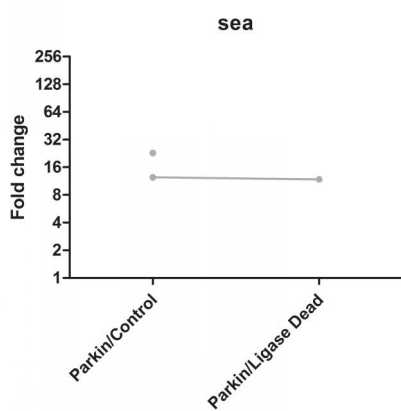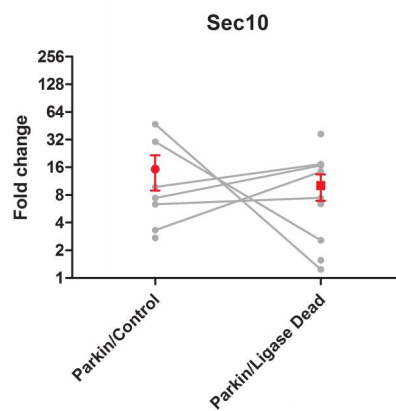

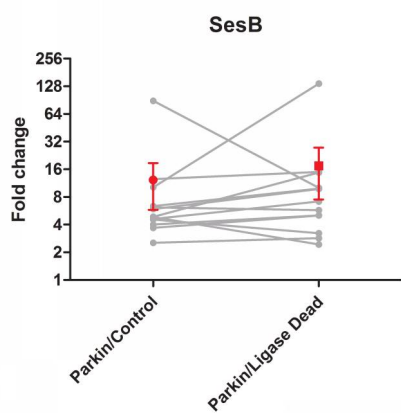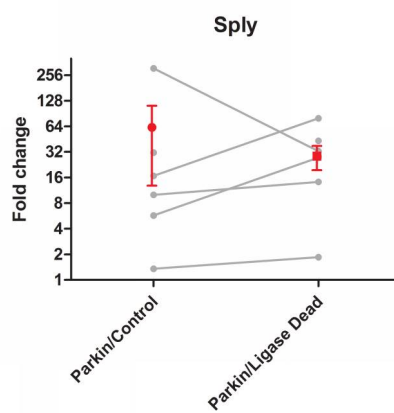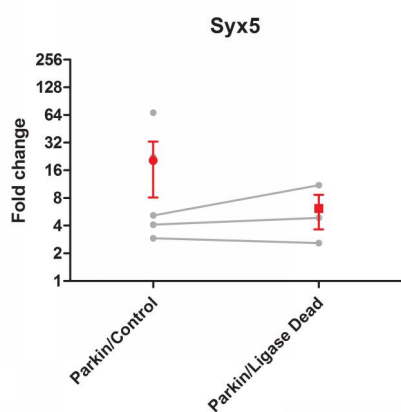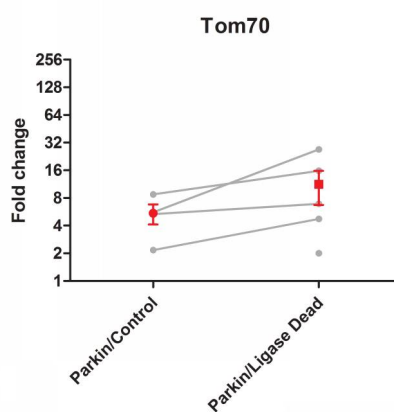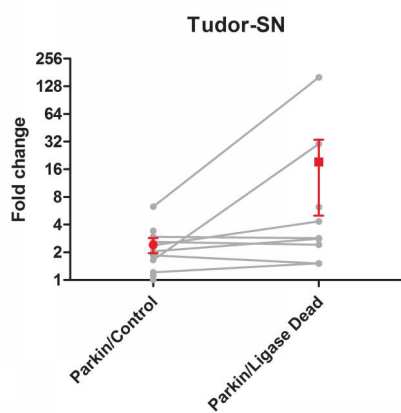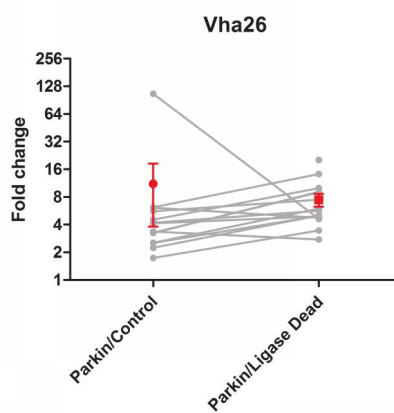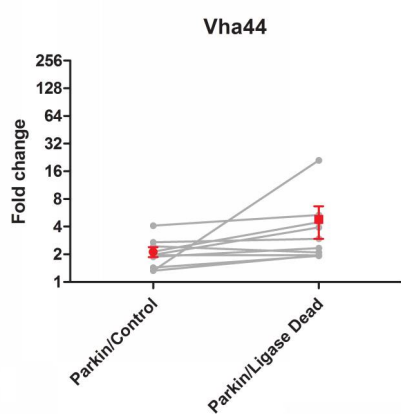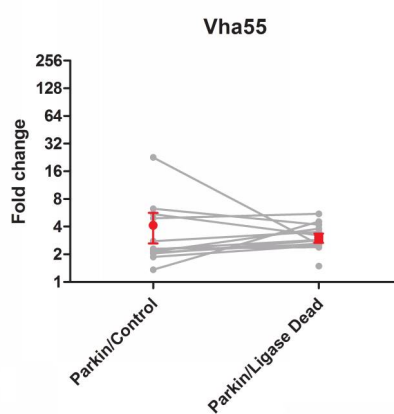

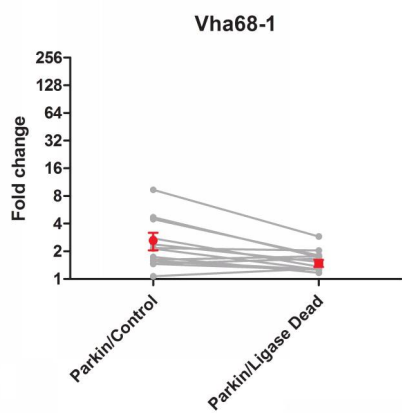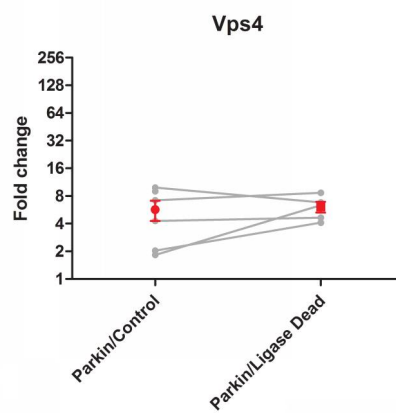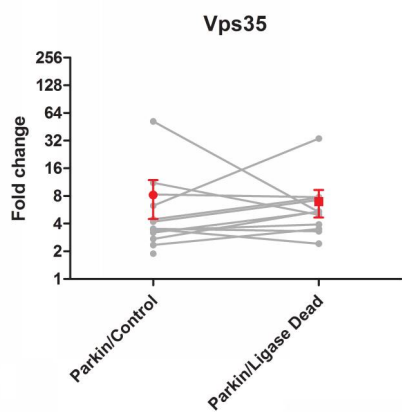

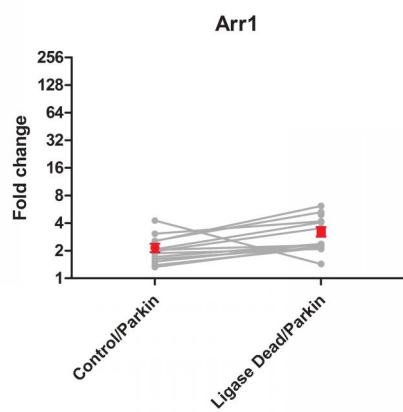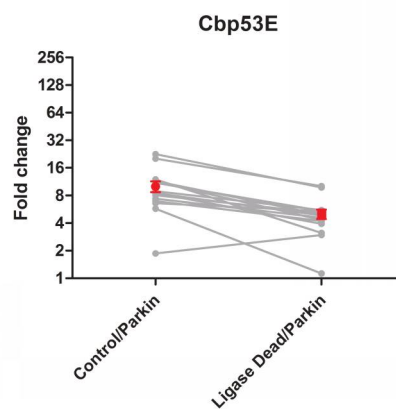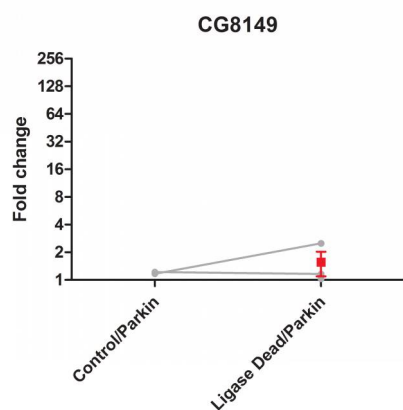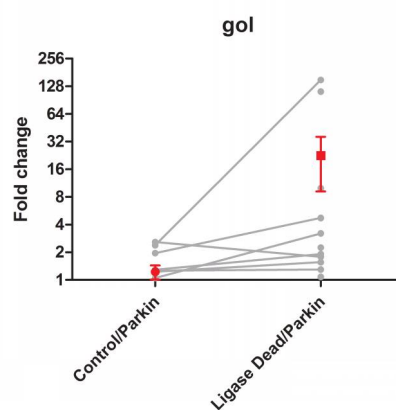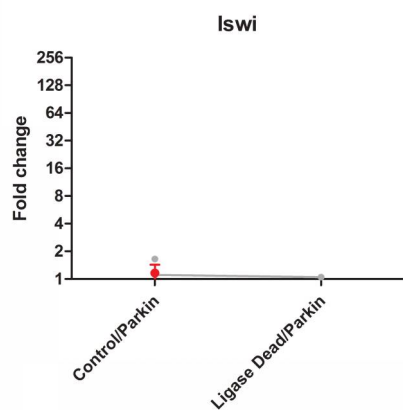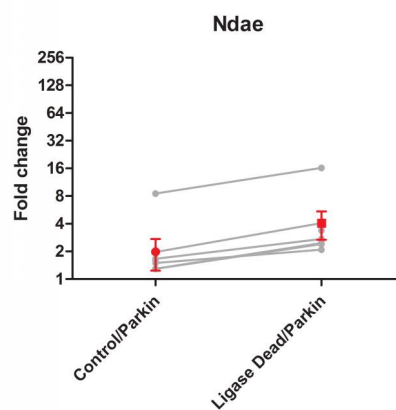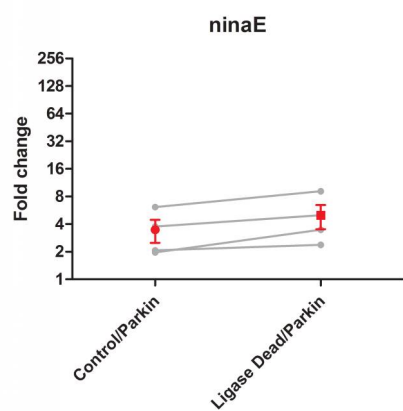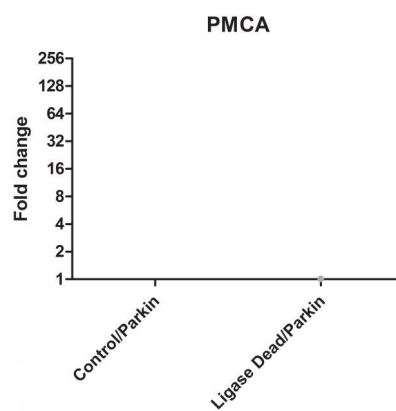

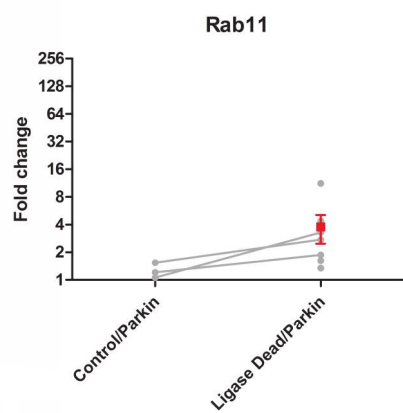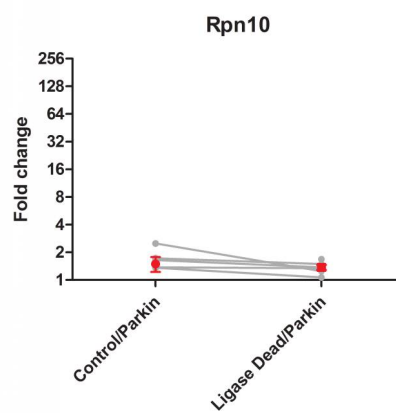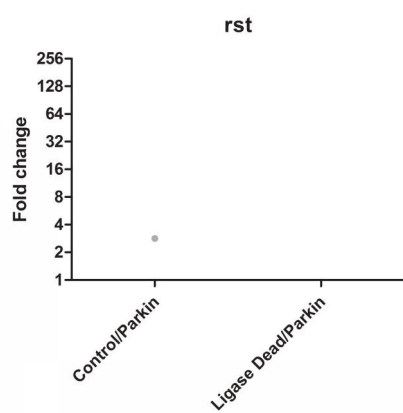

Supplementary Figure 5

Supplement: Supplementary file 5 — Peptide validation to identify the most robust Parkin responders. According to Perseus T-Test (Fold change >2, p-value < 0.05), 51 and 11 proteins were significantly more or less enriched respectively in purified ubiquitinated material of Parkin over-expressing head extracts, in a Parkin-dependent manner at the protein level with at least 2 unique peptides in all three relevant conditions (for enriched ParkinWT and for reduced ParkinLD). To further identify which are the ones that are more robustly associated to Parkin activity, the peptide profile of each of those proteins was analysed (see material and methods). A) Intensities of all the unique peptides in each condition (grey dots) and the average peptide intensity (red) are represented for each protein. When present, di-gly peptides are showed in blue. B) Fold change of all the unique peptides in each condition (grey dots) and the average fold change and standard error of the mean (SEM) are represented in red. Average intensity of the three replicates was used per condition and only peptides that appeared at least in two conditions were used. (PDF 3020 kb) [file 13024_2017_170_MOESM5_ESM.pdf]

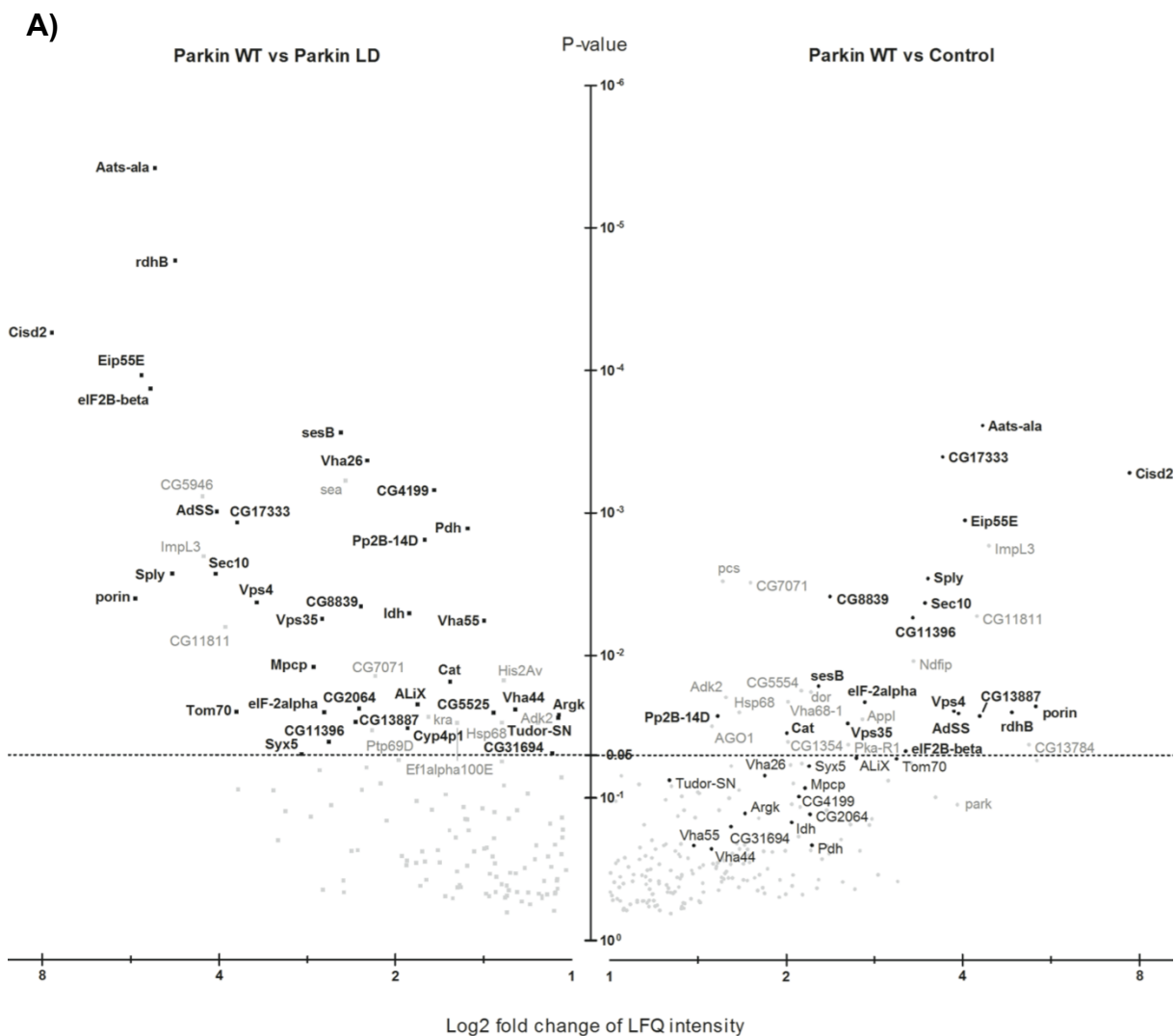

**B)** **Parkin<sup>WT</sup> vs Parkin<sup>LD</sup>**

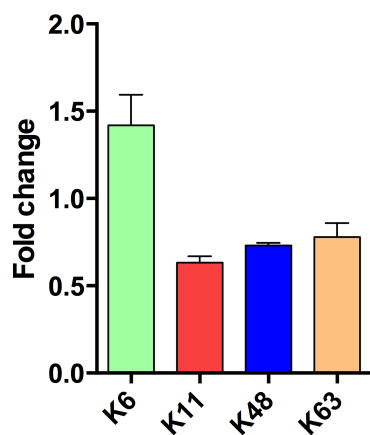

Supplementary Figure 6

Supplement: Supplementary file 6 — Proteins that are specifically more ubiquitinated after Parkin expression in two independent analyses and supplementary Ub chain graph. A) Outcome of two T-tests performed with Perseus are plotted in this volcano plot: ParkinWT vs ParkinLD (left side) and ParkinWT vs Control (BioUb; right side). Proteins are distributed along x axis by fold change of LFQ intensity and y axis by p-value. Proteins with a fold change bigger than 2 and a p-value lower than 0.05 are labelled with their names in grey. Proteins in bold, represent the most robust Parkin responders (see Table 1, Additional file 5: Figure S5). Fold change of LFQ intensity is represented in Log2 scale. B) C, Ub, WT, LD fly heads were subjected to BioUb pulldown and ubiquitin chains present in elutions were quantitated by comparison with isotopically labelled standards. Graph shows fold change of ubiquitin chain linkages found n ParkinWT vs ParkinLD. Error bars indicate standard deviation of three technical replicates. Complete genotypes of flies used: GMR-GAL4/CyO;UAS-BirA/TM6 (C), GMR-GAL4,UAS-( Bio Ub) 6 -BirA/CyO (Ub), GMR-GAL4,UAS-( Bio Ub) 6 -BirA/CyO;UAS-Parkin WT (WT) and GMR-GAL4,UAS-( Bio Ub) 6 -BirA/CyO;UAS-Parkin LD (LD). (PDF 414 kb) [file 13024_2017_170_MOESM6_ESM.pdf]

A

## Elution

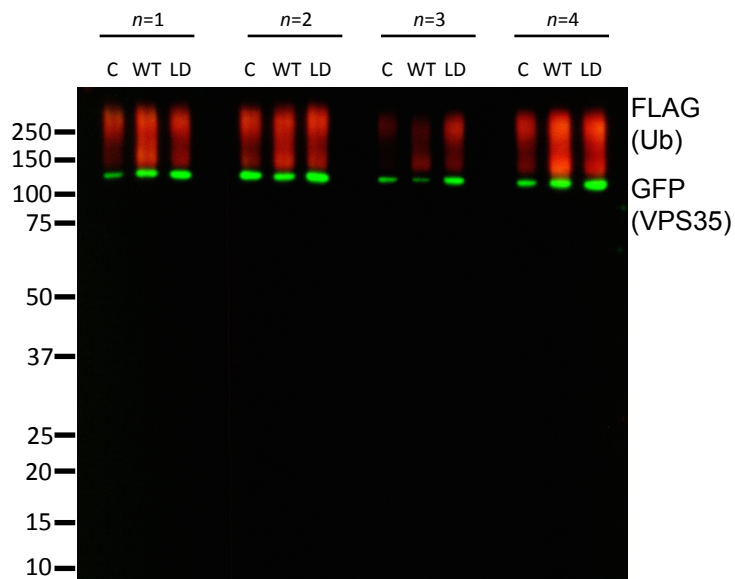

## Input

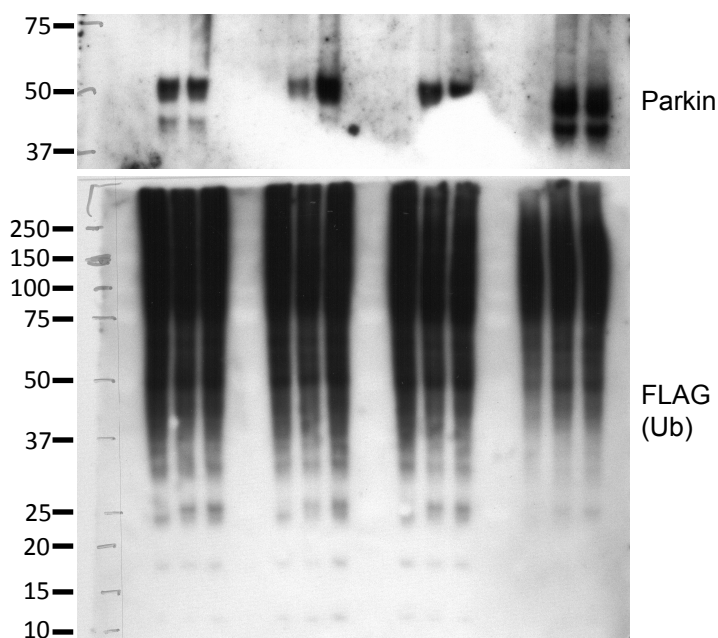

B

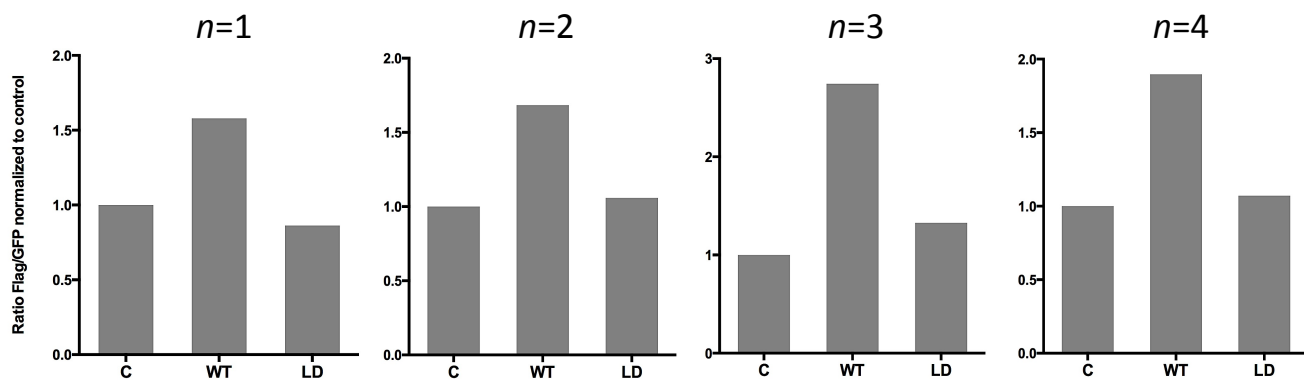

Supplementary Figure 7

Supplement: Supplementary file 7 — Over-expression of hParkin (WT) induced an increase of VPS35 ubiquitination compared to control (C) or to over-expression of inactive hParkin (LD) in four independent experiments. (A) The complete gels of Fig. 8a showing four independent in vivo ubiquitination assays for VPS35 in SH-SY5Y cells. Ubiquitination of YFP-tagged VPS35 was analysed by Western blot after capture of the YFP-tagged protein. Mouse anti-GFP antibody was used for detecting the captured VPS35 (shown in green), and HRP-conjugated anti-FLAG antibody for monitoring its ubiquitinated fraction (shown in red). Untagged Parkin over-expression levels in the whole cell extracts are monitored with anti-Parkin antibody. (B) Quantification of the ubiquitination status of VPS35 relative to the non-modified form was performed calculating the ratio FLAG:GFP with Image-J. The plot shows relative levels of VPS35 ubiquitination normalized to the GFP levels. (PDF 14700 kb) [file 13024_2017_170_MOESM7_ESM.pdf]
