# Supplementary material for: Quantitative proteomic analysis of Parkin substrates in Drosophila neurons
Source: Mol Neurodegener. 2017 Apr 11;12:29. doi: 10.1186/s13024-017-0170-3 (PMC5387213; doi:10.1186/s13024-017-0170-3)
Supplement: Supplementary file 8 — Alignment of Human and Drosophila Parkin. (PDF 61 kb) [file 13024_2017_170_MOESM8_ESM.pdf]

```
                              10        20        30        40        50        60
                               |         |         |         |         |         |
Human_O60260        --------------------------------------MIVFVRFNSSHGFPVEVDSDTS
Drosophila_Q7KTX7   MDYKDDDDKMSFIFKFIATFVRKMLELLQFGGKTLTHTLSIYVKTNGTKTLTVNLEPQWD
                                                          : ::*: *:.: :.*:::.: .

                              70        80        90       100       110       120
                               |         |         |         |         |         |
Human_O60260        IFQLKEVVAKRQGVPADQLRVIFAGKELRNDWTVQNCDLDQQSIVH-IVQRPWRKGQEMN
Drosophila_Q7KTX7   IKNVKELVAPQLGLQPDDLKIIFAGKELSDATTIEQCDLGQQSVLHAIRLRPPVQRQKIQ
                    * ::**:** : *: .*:*::******* :  *:::***.***::* *  **  : *:::

                             130       140       150       160       170       180
                               |         |         |         |         |         |
Human_O60260        ATGGDDPRNAAGGCEREPQSLTRVDLSSSVLPGDSVGLAVILHTDSRKDSPPAGSPAGRS
Drosophila_Q7KTX7   SATLE---------EEEP-SLS----DEASKPLNETLLDLQLESEERLNITDEERVRAK-
                    ::  :         *.** **:    ..:  * :.. * : *.::.* : .      .:

                             190       200       210       220       230       240
                               |         |         |         |         |         |
Human_O60260        IYNSFYVYCKGPCQRVQPGKLRVQCSTCRQATLTLTQGPSCWDDVLIPNRMSGECQSPHC
Drosophila_Q7KTX7   --AHFFVHCS-QCDKLCNGKLRVRCALCKGGAFTVHRDPECWDDVLKSRRIPGHCESLEV
                       *:*:*.  *:::  *****:*: *: .::*: :.*.****** ..*:.*.*:* .

                             250       260       270       280       290       300
                               |         |         |         |         |         |
Human_O60260        PGTS--------AEFFFKCGAHPTS-DKETSVALHLIATNSRNITCITCTDVRSPVLVFQ
Drosophila_Q7KTX7   ACVDNAAGDPPFAEFFFKCAEHVSGGEKDFAAPLNLIKNNIKNVPCLACTDVSDTVLVFP
                    . ..        *******. * :. :*: :..*:** .* :*:.*::**** ..****

                             310       320       330       340       350       360
                               |         |         |         |         |         |
Human_O60260        CNSRHVICLDCFHLYCVTRLNDRQFVHDPQLGYSLPCVAGCPNSLIKELHHFRILGEEQY
Drosophila_Q7KTX7   CASQHVTCIDCFRHYCRSRLGERQFMPHPDFGYTLPCPAGCEHSFIEEIHHFKLLTREEY
                    * *:** *:***: ** :**.:***: .*::**:*** *** :*:*:*:***::* .*:*

                             370       380       390       400       410       420
                               |         |         |         |         |         |
Human_O60260        NRYQQYGAEECVLQMGGVLCPRPGCGAGLLPEPDQRKVTCEGGNGLGCGFAFCRECKEAY
Drosophila_Q7KTX7   DRYQRFATEEYVLQAGGVLCPQPGCGMGLLVEPDCRKVTCQNG----CGYVFCRNCLQGY
                    :***::.:** *** ******:**** *** *** *****:.*    **:.***:* :.*

                             430       440       450       460       470       480
                               |         |         |         |         |         |
Human_O60260        HEGECS-AVFEASGTTTQAYRVDERAAEQARWEAASKETIKKTTKPCPRCHVPVEKNGGC
Drosophila_Q7KTX7   HIGECLPEGTGASATNSCEYTVDPNRAAEARWDEASNVTIKVSTKPCPKCRTPTERDGGC
                    * ***      **.*.:  * **  . * :***: **: *** :*****:*:.*.*::***

                             490       500       510
                               |         |         |
Human_O60260        MHMKCPQPQCRLEWCWNCGCEWNRVCMGDHWFDV
Drosophila_Q7KTX7   MHMVCTRAGCGFEWCWVCQTEWTRDCMGAHWFG-
                    *** *.:. * :**** *  **.* *** ***.
```

FLAG TAG: DYKDDDDK
Serine residue phosphorylated by PINK1: Ser65 (Human) and Ser94 (*Drosophila*)
Catalytic site cysteine: Cys431 (Human) and Cys449 (*Drosophila*)
UBL domain 1-76 (Human) and 30-106 (*Drosophila*)
RING0 domain 144-216 (Human) and 157-236(*Drosophila*)
RING1 domain 228-327 (Human) and 249-348(*Drosophila*)
IBR domain 328-377 (Human) and 349-394(*Drosophila*)
RING2 domain 415-465 (Human) and 433-482(*Drosophila*)

Supplementary Figure 8
